# Absolute Quantification of the Central Carbon Metabolome in Eight Commonly Applied Prokaryotic and Eukaryotic Model Systems

**DOI:** 10.3390/metabo10020074

**Published:** 2020-02-19

**Authors:** Lisa M. Røst, Lilja Brekke Thorfinnsdottir, Kanhaiya Kumar, Katsuya Fuchino, Ida Eide Langørgen, Zdenka Bartosova, Kåre Andre Kristiansen, Per Bruheim

**Affiliations:** Department of Biotechnology and Food Science; NTNU Norwegian University of Science and Technology, N-7491 Trondheim, Norway; lisa.m.rost@ntnu.no (L.M.R.); lilja.b.thorfinnsdottir@ntnu.no (L.B.T.); kanhaiya.kumar@ntnu.no (K.K.); katsuya.fuchino@ntnu.no (K.F.); ida.e.langorgen@ntnu.no (I.E.L.); zdenka.bartosova@ntnu.no (Z.B.); kare.a.kristiansen@ntnu.no (K.A.K.)

**Keywords:** tandem mass spectrometry, targeted metabolite profiling, central carbon metabolism, absolute quantification, intracellular metabolite pools, metabolome database, *B. subtilis*, *S. cerevisiae*, microalgae, human cell lines

## Abstract

Absolute quantification of intracellular metabolite pools is a prerequisite for modeling and in-depth biological interpretation of metabolomics data. It is the final step of an elaborate metabolomics workflow, with challenges associated with all steps—from sampling to quantifying the physicochemically diverse metabolite pool. Chromatographic separation combined with mass spectrometric (MS) detection is the superior platform for high coverage, selective, and sensitive detection of metabolites. Herein, we apply our quantitative MS-metabolomics workflow to measure and present the central carbon metabolome of a panel of commonly applied biological model systems. The workflow includes three chromatographic methods combined with isotope dilution tandem mass spectrometry to allow for absolute quantification of 68 metabolites of glycolysis, the pentose phosphate pathway, the tricarboxylic acid cycle, and the amino acid and (deoxy) nucleoside pools. The biological model systems; *Bacillus subtilis*, *Saccharomyces cerevisiae*, two microalgal species, and four human cell lines were all cultured in commonly applied culture media and sampled in exponential growth phase. Both literature and databases are scarce with comprehensive metabolite datasets, and existing entries range over several orders of magnitude. The workflow and metabolite panel presented herein can be employed to expand the list of reference metabolomes, as encouraged by the metabolomics community, in a continued effort to develop and refine high-quality quantitative metabolomics workflows.

## 1. Introduction

The field of mass spectrometry-based metabolome analysis, mass spectrometry (MS)-metabolomics, has developed remarkably over the last two decades. The resulting datasets have contributed to a more complete understanding of cell properties and responses in basic research, but also to applied fields such as biomarker discovery and metabolic engineering-driven optimization of cell factories [1,2,3,4,5]. Still, the number of publications presenting metabolite datasets to describe biological systems is rapidly increasing. The instrumentation, especially dominated by the liquid chromatography-mass spectrometry (LC-MS) platform, has become much more sensitive and robust, enabling high selectivity and throughput, and enhanced reproducibility. In addition, an impressive portfolio of software tools for advanced processing and interpretation has become available [6].

**Figure 1:**
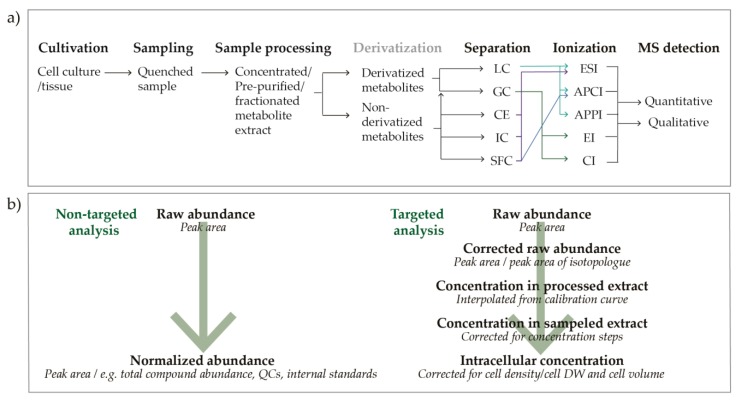
Mass spectrometry-based metabolomics workflow (**a**) from cultivation to detection, and (**b**) from raw abundance to normalized abundance (non-targeted analysis) or intracellular concentration (targeted analysis). LC, liquid chromatography; GC, gas chromatography; CE, Capillary electrophoresis; IC, Ion chromatography; SFC, Supercritical fluid chromatography; ESI, electrospray ionization; APCI, atmospheric pressure chemical ionization; APPI, atmospheric pressure photoionization; EI, electron ionization; CI, chemical ionization; MS, mass spectrometry; QC, quality control; DW, dry weight.

The metabolomics workflow is elaborate, with many steps requiring optimization and special attention, starting from reproducible cultivations, sampling and sample processing, to chromatographic separation and MS detection (Figure 1a), and finally, to data analysis and interpretation. The transformation from single-metabolite to comprehensive multi-metabolite analysis is not straightforward, and, contrary to the more well-established fields of genomics, transcriptomics, and proteomics, the workflow in the field of metabolomics is less standardized. The Metabolomics Society has addressed this by establishing several Scientific Tasks Groups [7]. Still, the metabolomics literature is dominated by single-lab protocols, as the analytical approach is heavily dependent on available MS instrumentation. To allow for comparison and broader application of available datasets, all labs would benefit from a generalized workflow. This is applicable both to non-targeted and targeted analysis, the two main strategies in metabolomics [8].

In this study, we focus on the ultimate goal of targeted metabolomics; reporting absolute intracellular metabolite concentrations. More specifically, we quantify the pathway intermediates of central carbon metabolism. These pathways are required for growth and reproduction in all organisms, leaving their intermediates amongst the most evolutionary conserved biomolecules [9]. As these central carbon metabolites carry the majority of metabolic flux, they are truly a depicter of cellular phenotype. The metabolomics community has recently encouraged focus on recording, mapping, and modeling of model organism metabolomes [10], including, but not limited to, central carbon metabolism. Though studies applying one or two analytical methods to report selected metabolite classes are available (e.g., [11,12,13]), few publications present comprehensive absolute concentration data [14]. Further, the discrepancy between reported concentrations, both in original literature and databases, is large. Reliable and reproducible absolute quantitative data is a prerequisite for successful application of kinetic and metabolite network modeling, but is to a large extent still lacking [15,16,17,18]. The fact that concentration entries in metabolite databases may vary over several orders of magnitude [19,20,21] is to be expected, as the database entries are recorded by different analytical methods, and from organisms cultivated at various conditions. The latter is crucial information, as both low-molecular metabolite and macromolecule composition of a cell is highly dependent on growth rate and physiological conditions [22,23,24]. Further, even with a strong focus on sampling and sample processing in the field, and many available publications [25,26,27], we are still left with compromised protocols for most biological model systems [28]. Even the long-time golden standard protocol for sampling yeast in cold methanol has been re-investigated and reported sensitive to a co-precipitation phenomenon, compromising its quantitative accuracy [29]. Another challenge in the field of metabolomics is the varying physicochemical properties of the metabolite classes, and the fact that some classes are extremely labile at certain conditions. This necessitates several metabolite class-specific sampling and analytical protocols applied in parallel to allow for broad coverage of the metabolome [30,31,32].

The main instrumental platform for MS targeted quantitative analysis is liquid chromatography -MS. Preferably, chromatographic separation combined with tandem mass spectrometric detection (MS/MS) to enhance separation, selectivity, and sensitivity [33]. Among the variety of available chromatographic separation techniques (Figure 1a), applicability varies with the physicochemical properties of the target metabolites. Reverse-phase (RP) LC is often the first choice due to its robust and reproducible performance, yet it fails for many low-molecular metabolite classes with highly charged metabolites, especially anions. The most frequently used alternative is hydrophobic interaction liquid chromatography (HILIC) [34]. However, both peak broadening, tailing, and a significant discrepancy between chromatographic behavior of analytical standards and actual biological extracts has been reported for this chromatographic technique [35,36]. For certain metabolite classes lacking selective precursor-product ion transitions (e.g. sugar phosphates), separation of single metabolites is strictly required. For this purpose, some labs still use gas chromatography (GC) or capillary electrophoresis (CE) separation [37,38]. Only a few labs apply Ion Chromatography (IC) on a routine basis, likely due to low throughput and high-maintenance instrumentation. Yet, it has superior resolution of phosphorylated metabolites [39,40,41]. For hydrophobic analytes such as long-chain fatty acids and lipids, supercritical fluid chromatography (SFC) is particularly well suited. Following separation, several modes of ionization are available other than the standard electrospray ionization (ESI). These can be tested for potentially enhancing sensitivity, if required for low abundant metabolites.

The next challenge in an MS-based targeted metabolomics workflow is absolute quantification; conversion from raw abundance to absolute intracellular concentration (Figure 1b). Ionization efficiency is concentration-dependent and highly sensitive to the influence of co-eluting matrix compounds. Thus, careful sample preparation, including crude purification steps, such as solid-phase extraction is recommended to improve the quality of the quantitative output [42]. In addition, the application of isotope dilution for correction is considered mandatory in targeted quantitative MS metabolite profiling [5,43,44,45]. Preferably, ^13^C/^15^N/deuterium-labeled isotopologues of all target metabolites should be applied. As commercial availability is limited, U^13^C-labeled biomass has been introduced [43] and is considered the present state-of-the-art. Next, corrected responses can be converted to extract concentrations by interpolation from the response of analytical standards. This is essential to allow for interpretation of relations between metabolites, such as ratios, as the ionization efficacy, and hence the response, varies between metabolites. Finally, intracellular concentrations can be calculated, given cell density/cell dry weight (DW) and cell volume. In sum, there are several potential pitfalls in an MS-metabolomics experiment. All steps need to be carefully optimized, and compromises must often be made to allow for a compatible workflow.

Herein, we present and apply our quantitative MS-metabolomics workflow to absolutely quantify intermediates of central carbon metabolism in a panel of popular biological model systems, including four microorganisms; *Bacillus subtilis*, *Saccharomyces cerevisiae*, *Nannochloropsis oceanica,* and *Phaeodactylum tricornutum,* and four human cell lines; Hek293, HeLa S3, MC/CAR, and NB4. Altogether, these organisms make up a panel with representatives of prokaryotes and eukaryotes, heterotrophs and phototrophs, unicellular organisms, and cells derived from multicellular organisms. Additionally, we have recorded metabolite profiles of *B. subtilis* and *S. cerevisiae* under different culture conditions, i.e., growing in both mineral and rich culture media. We apply three targeted tandem MS-methods to measure 68 metabolites of central carbon metabolism, including intermediates of glycolysis, the pentose phosphate pathway (PPP), and the tricarboxylic (TCA) acid cycle, and the amino acid and (deoxy) nucleoside phosphate pools. The workflow is laborious, yet we argue its necessity to allow the highest level precision and accuracy. All raw abundances are corrected by isotope dilution, applying the respective U^13^C/^15^N-isotopologue from a commercial vendor or a U^13^C-labeled metabolite extract of *S. cerevisiae*. Corrected abundances are converted to extract concentrations by interpolation from dilution series of analytical standards, and finally to intracellular concentrations applying measurements/estimations of biomass, cell DW, and cell volume. Trends and key findings are discussed in context of available data from metabolite databases and relevant literature. To our knowledge, this is the first report of absolute concentrations with high coverage of central carbon metabolism in a comprehensive panel of commonly applied biological systems.

## 2. Results and Discussion

### 2.1. Metabolite Pools of Central Carbon Metabolism Vary Over at Least Three Orders of Magnitude

**Figure 2:**
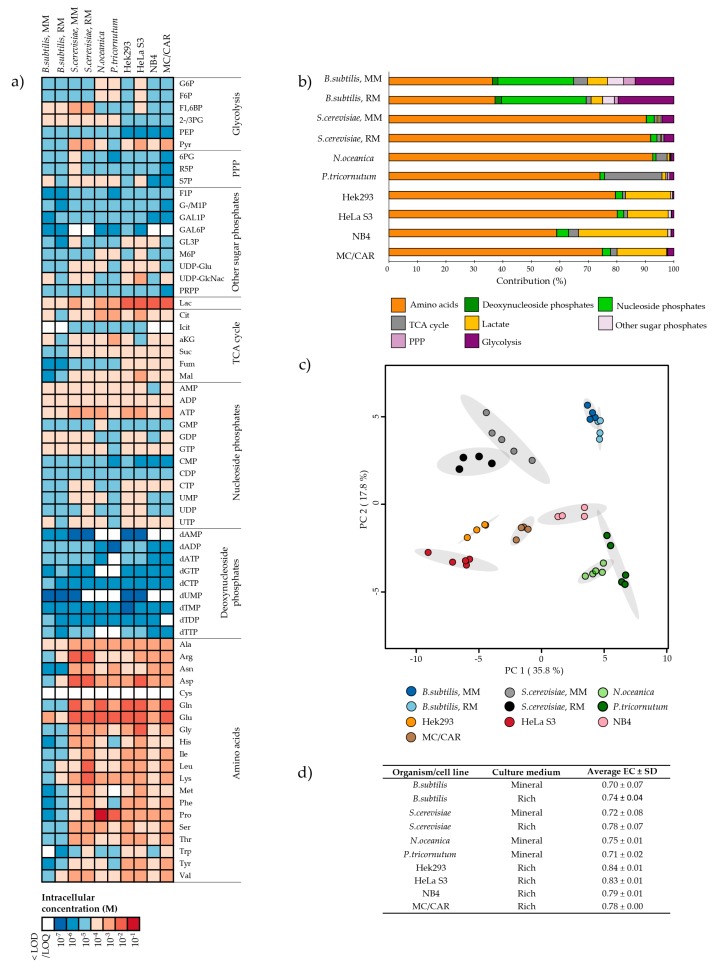
Intracellular metabolite levels in *Bacillus subtilis, Saccharomyces cerevisiae, Nannochloropsis oceanica, Phaeodactylum tricornutum,* and Hek293, HeLa S3, NB4, and MC/CAR cell lines. (**a**) Heat map of the average order of magnitude of intracellular concentrations (M), (**b**) contribution (%) of metabolite classes to the total measured level of metabolites, (**c**) scores plot from principal component analysis (PCA) of metabolite levels in all biological replicates with 95% confidence intervals [49], and (**d**) average energy charge (EC) ± standard deviation (SD). Rich media, RM; Mineral media, MM; TCA, tricarboxylic acid; PPP, pentose phosphate pathway. Metabolite abbreviations are listed in Table S1.

Comparing the average intracellular metabolite pool sizes of all eight biological systems revealed variation over at least three orders of magnitude in each organism/cell line, and seven orders of magnitude in the total panel (Figure 2a, Table S2). The majority of intracellular metabolite levels were in the micro- and millimolar concentration range, and the deoxynucleoside phosphates were the lowest abundant class in all species. However, while the level of amino acids by far exceeded all other metabolite classes in all eukaryotic species, it was lower and similar to the level of nucleoside phosphates in the prokaryote *B. subtilis* (Figure 2b). Not surprisingly, principal component analysis (PCA) revealed organism-specific clustering. The four human cell lines were separated along the first principal component (PC1), and from most other species along PC2. The two algal species clustered together. *S. cerevisiae* in mineral and rich media clustered together, yet only with slightly overlapping 95% confidence intervals. This also applied to *B. subtilis*. (Figure 2c). However, the applicability of such overall investigations is limited for this comprehensive quantitative dataset; thus, organism and cell line-specific metabolic features will be explored in further detail in separate sections. 

The adenylate energy charge (EC) of all biological systems was calculated from respective intracellular concentrations of the adenosine mono-, di-, and triphosphates AMP, ADP, and ATP (Figure 2d). Serving as a readout of the energy status of a cell [46], and hence the metabolite turnover, the experimental EC can be used to assess the quality of a sampling and quenching protocol. The EC of all organisms/cell lines of the panel was within the expected range of a physiological cell (0.7–0.95 [47]), confirming that the sampling protocols were tolerated. This is essential to ensure the reporting of physiologically relevant metabolite profiles. The EC of *B. subtilis* and *S. cerevisiae* was slightly higher when cultured in rich compared to mineral media, indicating a greater energy reserve of the former. The EC of adherent cell lines was the highest in the panel. However, this might be attributed to the nature of their sampling protocol, employing mechanical detachment rather than filtering. 

Interpretation of metabolite data is challenging, as there is no direct proportionality between pool sizes and intracellular metabolic fluxes [48]. Quite contrary, the accumulation of end product in a linear pathway will downregulate the pathway flux through feedback inhibition. Central metabolic pathways such as glycolysis, the PPP, and the TCA cycle are regulated in a complex manner to both serve complete oxidation of carbon sources for maximum yield of energy, and to supply carbon for macromolecule building blocks. Consequently, these pathways cannot simply shut down when growth ceases. Additionally, microorganisms in particular adapt growth rates and metabolism in response to cultivation conditions. This also includes macromolecular compositions, as introduced through seminal papers five to six decades back, e.g., Schaechter and co-workers demonstrated that the RNA and protein content of *Salmonella typhimurium* increased and decreased with increasing growth rate, respectively [24]. Thus, interpretation of metabolite data must be placed in a biological context where both growth rate and the rates of substrate uptake and product excretion are reported and considered. A thorough exploration of growth rate-, medium composition-, and cultivation condition-dependency of the metabolome is beyond the scope of this study.

### 2.2. Intracellular Metabolite Levels in Bacillus subtilis are Highly Dependent on Culture Medium Composition

*B. subtilis* has become the most studied species of gram-positive bacteria and is largely exploited as a microbial cell factory [50]. The full *B. subtilis* genome was sequenced in 1997 [51], and its annotation is reported in several dedicated databases, such as ‘SubtiWiki’ and ‘BSubCyc’. Yet, to our knowledge, no database listing intracellular metabolite concentrations of this species is publicly available. Hence, the metabolite profile of *B. subtilis* will be explored through general comparisons between prokaryotes and eukaryotes, and mineral versus rich media.

The relative contribution of metabolite classes to the total pool of ‘panel metabolites’ cannot be interpolated from Figure 2a, as it is not taking the total intracellular concentration of each species into account. However, comparing the relative contribution of different metabolite classes (Figure 2b, Table S3), the low amino acid and high nucleoside phosphate levels in *B. subtilis* stand out. While the amino acids alone constitute ~60–90% of the profiled metabolites in all eukaryotic species, it only constitutes ~37% in *B.* subtilis. Inversely, while nucleoside phosphates constitute ~26–30% of the *B. subtilis* metabolites, it constitutes only 1–4% in all profiled eukaryotes. To some extent, these observations may be correlated with differences between average prokaryotic and eukaryotic macromolecule composition and regulation strategies. Rapidly growing cells has a high content of RNA, as first described by Schaechter [24], and further; while prokaryotes employ rapid mRNA-turnover to regulate protein synthesis [52], eukaryotes rely on post-translational regulation mechanisms to a larger extent [53].

**Figure 3:**
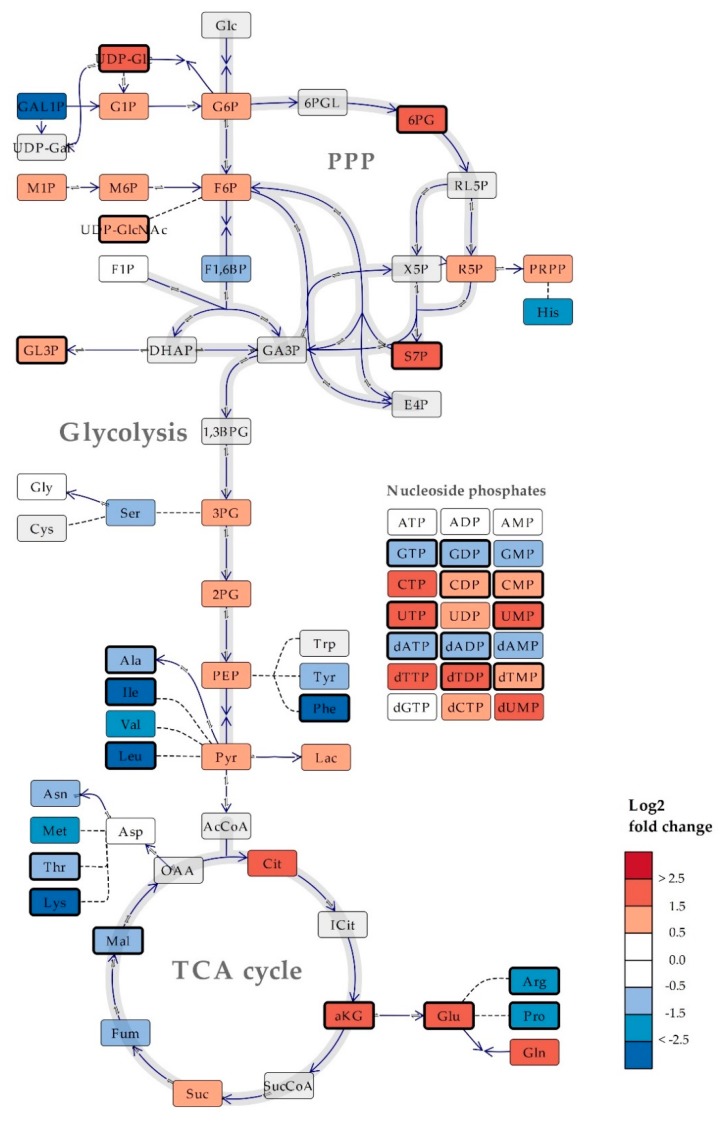
Simplified schematic overview of central carbon metabolism in *Bacillus subtilis,* with heat map of the log_2_ fold change of average metabolite levels in cells cultured in mineral versus rich media. Bold lined rectangles indicate statistically significant metabolite levels (Two-tailed T-test, adjusted *p*-value (False Discovery Rate (FDR)) = 0.05). Levels of metabolites colored dark grey were < limit of quantification/detection. Metabolites colored light grey were not measured. Direct reactions are indicated by continuous lines, a series of reactions are indicated by dashed lines. Co-enzymes and -substrates are not included. TCA, tricarboxylic acid; PPP, pentose phosphate pathway; Glc, glucose; 6PGL, 6-phosphogluconic acid; RL5P, ribulose 5-phosphate; X5P, xylulose 5-phosphate; UDP-gal, UDP-galactose; DHAP, dihydroxyacetone phosphate; GA3P, glyceraldehyde 3-phosphate; 1,3BPG, 1,3-bisphosphoclyceric acid; AcCoA, Acetyl-CoA; SucCoA, Succinyl-CoA; OAA, oxaloacetic acid. Abbreviations for quantified metabolites are listed in Table S1.

Along the line of ‘the growth law’ for macromolecules, as introduced by Schaechter [24], the low-molecular metabolite pool composition of *B. subtilis* could also vary between the tested culture conditions, considering the experimental generation time of 30 and 78 min in rich and mineral media, respectively. While the relative distribution of metabolite classes only revealed subtle differences (Figure 2b), the close to non-overlapping confidence intervals of the PCA scores plot (Figure 2c) indicate that the two are indeed different. Increasing resolution to the single metabolite level revealed significant (*p* = 0.05) differences in metabolite pools of all metabolic pathways (Figure 3). The intracellular pool sizes of glycolysis, PPP, and the first half of the TCA cycle were higher in *B. subtilis* in mineral media compared to the rich media, and inversely, the concentrations of most amino acids were lower. One marked exception was the four-fold higher levels of the abundant amino acids glutamic acid (Glu) and glutamine (Gln), leaving the size of the total amino acid pool similar to that of *B. subtilis* in rich media, as shown in Figure 2b. The higher intracellular pools of α-Ketoglutaric acid (aKG), Glu, and Gln might reflect the nutrient availability in mineral media. As the mineral media does not contain an organic source of nitrogen, *B. subtilis* must assimilate reduced nitrogen from the medium into amino acids and other nitrogen-containing metabolites to grow. The aKG, Glu, and Gln provide the critical entry point of nitrogen, the two latter serving as donors for further transamination reactions [54].

### 2.3. Several Metabolic Features are Conserved Across Saccharomyces cerevisiae and Bacillus subtilis Cultured in Mineral Compared to Rich Media

**Figure 4:**
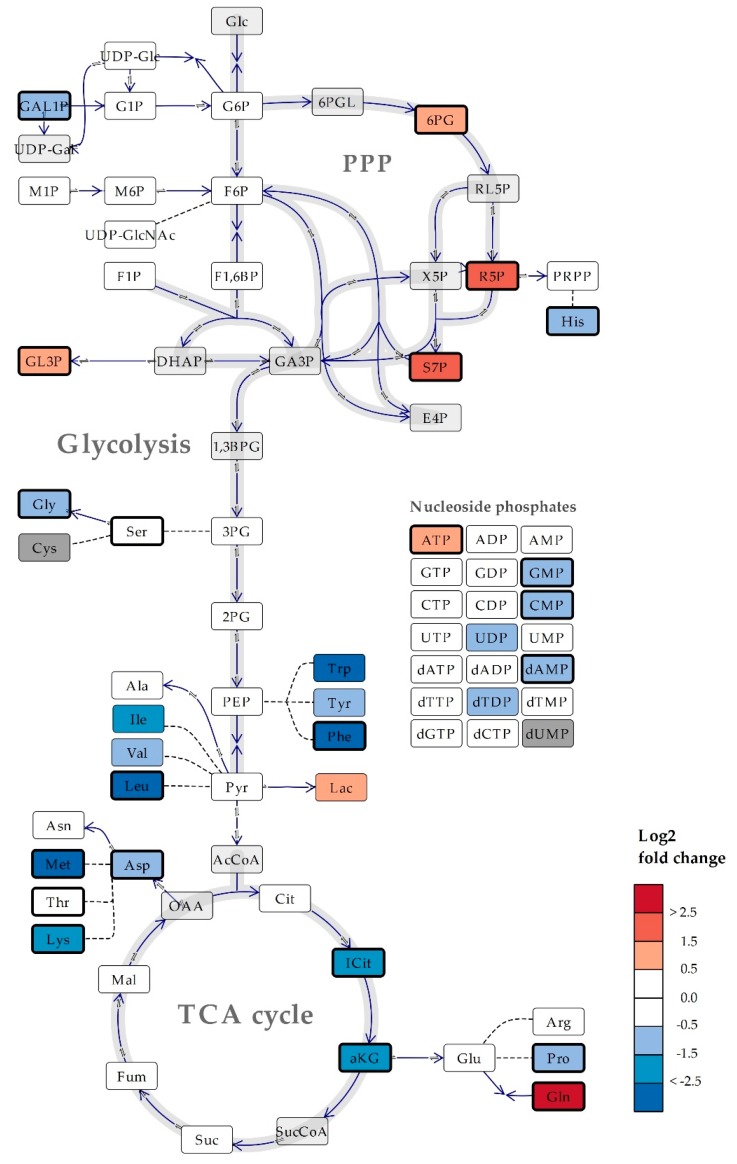
Simplified schematic overview of central carbon metabolism in *Saccharomyces cerevisiae*, with heat map of the log_2_ fold change of average metabolite levels in cells cultured in mineral versus rich media. Bold lined rectangles indicate statistically significant metabolite levels (Two-tailed T-test, adjusted *p*-value (FDR) = 0.05). Levels of metabolites colored dark grey were < limit of quantification/detection. Metabolites colored light grey were not measured. Direct reactions are indicated by continuous lines, a series of reactions are indicated by dashed lines. Co-enzymes and -substrates are not included. TCA; tricarboxylic acid, PPP, pentose phosphate pathway; Glc, glucose; 6PGL, 6-phosphogluconic acid; RL5P, ribulose 5-phosphate; X5P, xylulose 5-phosphate; UDP-gal, UDP-galactose; DHAP, dihydroxyacetone phosphate; GA3P, glyceraldehyde 3-phosphate; 1,3BPG, 1,3-bisphosphoclyceric acid; AcCoA, Acetyl-CoA; SucCoA, Succinyl-CoA; OAA, oxaloacetic acid. Abbreviations for quantified metabolites are listed in Table S1.

Compared to the prokaryote *B. subtilis*, the influence of culture medium on intracellular metabolite levels was significant, but less comprehensive in the eukaryotic model organism *S. cerevisiae* (Figure 4), which was also able to sustain its growth rate in the mineral medium. Notably, some features were conserved across both organisms; the concentrations of most amino acids were significantly lower in mineral compared to rich media, yet the level of Gln was higher. As discussed for *B. subtilis*, the elevated Gln levels might indicate enhanced nitrogen assimilation required in the absence of a source of organic nitrogen. Further, all measured intermediates of the PPP were significantly lower in both species cultured in mineral compared to rich media. This depletion might be attributed to the need for de novo synthesis of nucleosides from ribose 5-phosphate (R5P) which could be salvaged from rich media, and along that line, the need for reduced nicotinamide adenine dinucleotide phosphate (NADPH) to sustain anabolic reactions.

The Yeast Metabolome Database (YMDB) is a database dedicated to the metabolome of *S. cerevisiae* [21]. As of January 2020, it contains 16042 entries on small molecule metabolites in yeast, collected from textbooks, scientific journals metabolic reconstructions, and other electronic databases. Hence, both growth medium and conditions, sampling procedure, sample processing, analytical methodology, and correction strategies may vary between metabolite concentration entries, and consequently, some entries range over several orders of magnitude. Comparing the intracellular metabolite levels in our *S. cerevisiae* panel to respective YMDB entries revealed incomplete coverage, even with respect to central carbon metabolism. As much as 41% of our panel lacked entries of intracellular concentration in YMDB. In particular, the PPP and nucleoside phosphate pools were poorly covered (Table S4). Out of the remaining 59%, most metabolite levels reported in our panel was within, or close to, the (sometimes very large) range of corresponding YMDB entries. Discrepancies were recorded for certain amino acids, but the most conspicuous difference was the two first intermediates of glycolysis; glucose 6-phosphate and fructose 6-phosphate. The entered (sub)-millimolar concentrations of these metabolites were measured by spectroscopic methods in the 1980s [55], at concentrations 10 to 100 times higher than our dataset. More recent data might be available, but nevertheless, these YMDB entries highlight the need for repeated measurements with more accurate and precise analytical methodology, as presented in this study.

### 2.4. The Microalgae Nannochloropsis oceanica and Phaeodactylum tricornutum are Both High in Proline

Two sequenced model microalgal species, *N. oceanica* [56] and *P. tricornutum* [57], were included in the panel for metabolite profiling. Due to ease of genetic manipulation and high yield of nutritionally beneficial polyunsaturated fatty acids, these species are gaining interest for industrial applications [56,58,59].

As reviewed in [60], extracting microalgae requires special attention due to their rigid cell wall. To balance complete extraction with conservation of labile metabolites, we did not consider ‘high energy-input methods’ such as heating nor wave energy. Rather, complete removal of the green whole-cell pellet was obtained by extending the extraction protocol employed for the other organisms from three to six repeated freeze-thaw cycles. The resulting EC of both microalgae species was comparable to the EC recorded for the other organisms (Figure 2d), indicating that this extension was tolerated.

**Figure 5:**
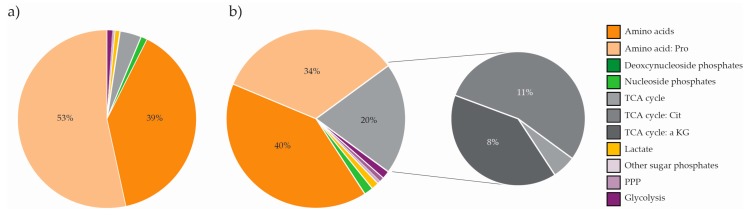
Contribution (%) of metabolite classes to the total measured level of metabolites in microalgae (**a**) *Nannochloropsis oceanica* and (**b**) *Phaeodactylum tricornutum,* including the contribution of TCA cycle intermediates to the total measured TCA cycle level in *P. tricornutum*. Contributions > 4% are indicated with numbers. TCA, tricarboxylic acid; PPP, pentose phosphate pathway; Pro, proline; Cit, citrate; aKG, α-ketoglutaric acid.

Out of all metabolites profiled across all species in this study, proline (Pro) stands out as the only metabolite present in a sub-molar concentration, as measured in *N. oceanica* (10^−1^ M, Figure 2a, Table S2). Though slightly lower in *P. tricornutum* (10^−2^ M), the concentration of Pro in microalgae exceeded that of all other profiled species by at least one to two orders of magnitude. In fact, Pro alone constituted 34% and 54% of the total profiled pool in *N. oceanica* (Figure 5a) and *P. tricornutum* (Figure 5b), respectively. The high levels of Pro in these species are in accordance with published literature on both Nannochloropsis species and several other species of microalgae, including *P. tricornutum*; reporting Pro as an important compatible osmoprotecting solute in microalgae [61,62].

Additionally, the high proportion of TCA cycle intermediates in *P. tricornutum* stood out from the rest of the panel (Figure 2b). This could mostly be attributed to high levels of Citrate (Cit) and aKG (Figure 5b, right panel). Other studies have implicated the TCA cycle and accumulation of Cit, specifically in the response to nutrient stress in this species [58,63], indicating that flux to and from Cit might be an important regulatory node in *P. tricornutum*.

### 2.5. The Metabolite Profile of Human Cell Lines Varies with Tissue of Origin

Cell lines have emerged as an invaluable scientific tool, allowing for studies of animal and human biology, disease, and response to stressors in a controlled environment without the ethical implications of studying whole tissue or organisms. We included a clone of the first ever established and most commonly employed human cell line; HeLa [64], the popular mammalian protein expression system Hek293, and two hematological cancer cell lines of different cell type origin in our panel for metabolite profiling, serving as representatives of different tissue and cell types. High intracellular levels of lactic acid (Lac) made all four cell lines stand out from the panel of microorganisms (Figure 2a). In fact, Lac alone made up 14–31% of the total measured pool in cell lines, compared to ~ 0.5% in the eukaryote *S. cerevisiae* (Figure 2b, Table S3). This phenomenon, termed ‘aerobic glycolysis’; high yield of Lac even in the presence of oxygen, is a well-known feature of cancer metabolism, first described by Otto Warburg in 1924 [65]. The uncoupling of glycolysis from downstream oxidative phosphorylation is inefficient in terms of generating ATP. However, it is recognized to scavenge carbon for precursors of macromolecular synthesis required for cell division, justifying this phenotype in rapidly proliferating tissue such as the cell lines of the panel [66]. As for the other eukaryotic species of the panel, high levels of Glu and Gln stood out in all human cell lines. This likely reflects the key roles of these metabolites in nitrogen assimilation and transamination reactions, as previously discussed.

Looking beyond the common high intracellular levels of Lac, Glu, and Gln, we found the total level of metabolites measured to be very different amongst most cell lines, even after normalization to experimental cell density and cell volume. The total level of metabolites in NB4, a cancer of myeloid cells isolated from bone marrow, was half of the total level of metabolites in MC/CAR, a cancer of plasma cells isolated from peripheral blood. Further, MC/CAR metabolite levels were half of those of HeLa S3 and Hek293, originating from tissue of the cervix and embryonic kidney, respectively (Table S3). The different total metabolite levels of the hematological cancers, both sampled by filtering, indicates that this feature cannot simply be attributed to sampling procedure (filtering vs. mechanical detachment).

**Figure 6:**
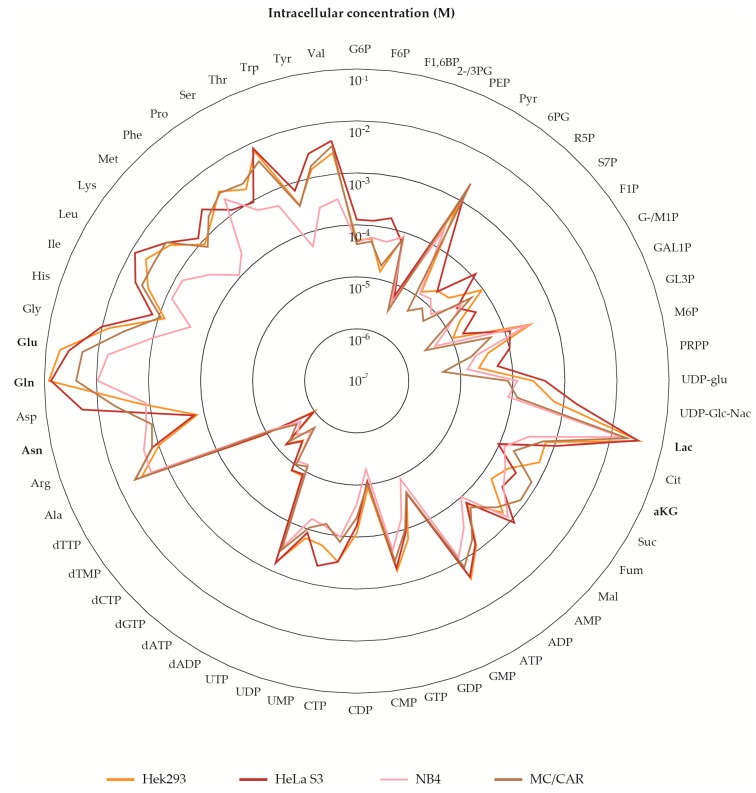
Log_10_ -transformed average metabolite pool sizes of the Hek293, HeLa S3, NB4, and MC/CAR cell lines. Metabolites in bold are mentioned in the text. Metabolite abbreviations are listed in Table S1.

The separation of cell lines (Figure 2c) persisted when re-calculating principal components for the cell lines only (Figure S1a). The PCA loadings (Figure S1b) and graphing the intracellular levels of all panel metabolites (Figure 6) revealed that separation was not solely attributed to different total levels, e.g., HeLa cells, having the highest total level, had the lowest levels of aKG, a key metabolite located at the intersection of carbon and nitrogen metabolism. The aKG serves regulatory roles far beyond nitrogen assimilation and energy generation [54]. We refrain from speculations on the implications of the various aKG levels detected in our panel, which would be particularly challenging for this key regulatory metabolite. Another marked example is NB4, having the lowest total level of metabolites, and remarkably low amino acid levels compared to the other cell lines (See also Figure 2b). Yet, NB4 asparagine (Asn) levels were ten times higher than those of cell lines from solid tissue. Asn is required for the synthesis of glycoproteins, allowing for N-linked glycosylation. As glycoproteins play key roles in immune cells, the particularly high levels of Asn measured in both NB4 and MC/CAR might reflect the specialized roles of these cell types.

The recorded differences, both in levels of specific and total metabolites emphasizes the importance of accounting for cell type-specific metabolite profiles in biological studies. The recording of the human metabolome, as collected in The Human Metabolome Database (HMDB) is extensive, containing more than 100,000 metabolite entries [19]. Yet, as the YMDB, this database is still far from complete. Though all metabolites in our panel of glycolytic and PPP intermediates were entered for body fluids, recordings of intracellular levels were scarce, in fact just collected from one publication describing one specific cell type [67]. The detected differences in the cell lines of our panel indicate that the metabolite levels of one cell type are not necessarily representative of metabolite levels in another cell type. This further demonstrates the need for increasing the number of reports on absolute intracellular concentrations of various organisms and cell types to strengthen metabolome databases as a reference tool.

### 2.6. Concluding Remarks

During the course of this study, we experienced the need for adjusting, optimizing, and validating each step of the metabolomics workflow (Figure 1a) for each individual biological system. We strongly recommend not to uncritically apply published protocols, especially for sampling and sample processing. Searching the literature for filtering protocols, we found many publications to incompletely report sampling parameters. To allow for inter-lab validation and broader application of available protocols and datasets, we encourage detailed reporting of sampling parameters, including a measure of cell density at the time of sampling (OD, dry weight/l or cell density), a measure of the number of cells sampled per filter, and a parameter applied to estimate the robustness of the sampling protocol, such as the EC. The widely applied model organism *Escherichia coli* was excluded from our panel for metabolite profiling after initial rounds of testing, as we did not measure a satisfactory EC nor rinsing of extracellular metabolites by applying filtering protocols based on available information in published literature. The sampling parameters and resulting EC of three tested methods are included in Supplementary Table S5, clearly demonstrating that varying sampling parameters can have a marked effect on the EC, and that sampling by cold centrifugation is not an option for the high-turnover nucleoside phosphates. The fact that we could not simply transfer the sampling protocol of e.g., *S. cerevisiae* to *E. coli*, emphasize the importance of tailoring a sampling protocol to the properties of the biological system. As illustrated by the necessity of extending the extraction protocol for microalgal species, this also applies to extraction. When analyzing eight different biological systems, we also experienced the need to adjust downstream analysis, i.e., applying differently balanced standard mixtures, and even apply transitions with different sensitivity measuring Lac in human versus microbial systems. This again demonstrates the importance of adapting and optimizing a generalized metabolomics workflow for each model species.

As previously mentioned, both the YMDB and HMDB were incomplete with regard to coverage of our panel of metabolites. These databases can serve as important reference tools for validating new methods given comprehensiveness and analytical precision. Not to mention, such data repositories are required for kinetic and metabolite network modeling. Contrary to the well-established fields of genomics and transcriptomics, metabolomics data repository is not standardized. A set of requirements should follow with reporting these data to account for possible sources of variation, including physiological state, cultivation conditions, sampling, and sample processing protocols. Importantly for MS-based analyses, isotope dilution should always be applied for the correction of experimental and analytical variation, to ensure the highest level of accuracy and precision. Further, we suggest recording not only total dry weight, but dry weight and volume of single cells, to allow for standardized reporting of intracellular concentrations (Figure 1b).

Given the limited basis for comparison with databases, it is hard to judge the dataset presented herein by other criteria than statistical measures and EC. Yet, detection of recognized metabolic features of the biological systems of the panel, such as high levels of Pro and Lac in microalgae and proliferating human cells, respectively, substantiates the reporting of biologically significant levels and trends. Further, the statistically significant differences between mineral and rich media mark the importance of standardized culturing conditions for measuring and reporting the ‘reference metabolome’ of model organisms, as encouraged by the metabolomics community [10]. Furthermore, it invites the community to perform follow-up studies on metabolome variation and dependencies, as both *B. subtilis* and *S. cerevisiae* are important microbial cell factories.

## 3. Materials and Methods 

### 3.1. Cultivation 

#### 3.1.1. Cell Lines

The adherent cell lines Hek293 (Human embryonic kidney, ATTC^®^ CRL-1573, ATCC, Manassas, VA, USA) and HeLa S3 (Cervical cancer, ATTC^®^ CCL-2.2) were cultured in DMEM high glucose (D6429, Sigma-Aldrich, St. Louis, MO, USA). The suspension cell lines NB4 (Acute promyelocytic leukemia, kindly gifted by Professor Stein Døskeland, University of Bergen, Bergen, Norway [68]) and MC/CAR (Myeloma, ATTC^®^ CRL-8083) were cultured in RPMI-1640 (R8758, Sigma-Aldrich) and IMDM (21980-032, Thermo Fisher Scientific, Waltham, MA, USA) media, respectively. All culture media were supplemented with 2 mM glutamine (K0283, VWR, Radnor, PA, USA), 100 µg/mL gentamicin (G1272, Sigma-Aldrich), 2.5 µg/mL amphotericin (A2942, Sigma-Aldrich), and fetal bovine serum (F7524, Sigma-Aldrich), 10% in DMEM high glucose and RPMI-1640, and 20% in IMDM. All cell cultures were maintained at 37 °C in a humidified atmosphere of 5% CO_2_.

#### 3.1.2. *Bacillus subtilis*

Parallel cultures of *B. subtilis* (lab strain) were cultured in rich or mineral media in baffled flasks. Flasks were incubated at 37 °C with continuous stirring at 200 rpm. The rich media was prepared from 10 g/L tryptone (T9410, Sigma-Aldrich), 10 g/L NaCl (27810.295, VWR) and 5 g/L yeast extract (92144, Sigma-Aldrich). The mineral media was prepared in MilliQ-H_2_O (MQ-H_2_O) by dissolving 11.2 g/L Na_2_HPO_4_-7H_2_O (S9390, Sigma-Aldrich), 3 g/L KH_2_PO_4_ (P5655, Sigma-Aldrich), 0.5 g/L NaCl (27810.295, VWR), 0.5 g/L NH_4_Cl (A9434, Sigma-Aldrich), 0.2465 g/L MgSO_4_-7H_2_O (M5921, Sigma-Aldrich), 0.1470 g/L CaCl2-2H_2_O (223506, Sigma-Aldrich), 4 g/L glucose (101176K, VWR) and 1 mL/L media of a trace element solution containing 10 g/L FeSO_4_-7H_2_O (F8633, Sigma-Aldrich), 2.25 g/L ZnSO_4_-7H_2_O (Z0251, Sigma-Aldrich), 2 g/L CaCl_2_-2H_2_O (223506, Sigma-Aldrich), 1 g/L CuSO_4_-5H_2_O (197722500, Thermo Fisher Scientific), 0.38 g/L MnCl_2_-4H_2_O (M5005, Sigma-Aldrich), 0.14 g/L H_2_BO_3_ (B6768, Sigma-Aldrich), and 0.1 g/L (NH_4_)_6_Mo_7_O_24_-4H_2_O (1011820250, Merck Millipore, Damstadt, Germany). The final media was supplemented with 600 µg/L CoCl_2_-6H_2_O (33606, VWR), 1 mg/L biotin (47868, Sigma-Aldrich), and 1 mg/L thiamine hydrochloride (T1270, Sigma-Aldrich).

#### 3.1.3. *Saccharomyces cerevisiae*

Parallel cultures of *S. cerevisiae* (CEN.PK 113-7D) were cultured in rich or mineral media in baffled flasks. Flasks were incubated at 30 °C with continuous stirring at 200 rpm. The rich media was prepared from 50 g/L YPD broth (Y1375, Sigma-Aldrich) in MQ-H_2_O and autoclaved. The mineral media was prepared by dissolving 6.8 g/L YNB (Y0626, Sigma-Aldrich) and 5 g/L glucose (101176K, VWR) in MQ-H_2_O, and sterile filtering the complete medium.

#### 3.1.4. *Nannocloropsis oceanica* and *Phaeodactylum tricornutum*

Parallel axenic cultures of *N. oceanica* (CCMP1779) and *P. tricornutum* (clone Pt1 8.6, CCMP632) were cultured in 75 cm^2^ culture flasks (734-2313, VWR) in f/2 media [69] prepared from filtered natural sea water from the Trondheim fjord. Flasks were incubated at 20°C with continuous stirring at 170 rpm under continuous cool white fluorescent light of ~35 µmol photons m^−2^ s^−1^.

### 3.2. Sampling

#### 3.2.1. Suspension Cell Lines

Five replicate cultures of the suspension cell lines NB4 and MC/CAR were seeded in 75 cm^2^ culture flasks (734–4139, VWR) and incubated overnight to reach a density of ~5.0 × 10^5^ cells/mL. Exponentially growing cells were sampled according to a filtering protocol adapted from [27]. 5 mL aliquots of cell suspension were fast filtered at a vacuum pressure 250 mbar below the ambient pressure and harvested on hydrophilic polyvinylidene fluorine filters with a pore size of 5 µm (SVLP04700, Merck Millipore). Cells were rinsed for residual medium with 10 mL cold saline and 10 mL cold MQ-H_2_O. Two filters were pooled into a centrifuge tube containing 13 mL of MQ-H_2_O:Acetonitrile (ACN, 1:1, *v/v*), quenched in liquid nitrogen (LN_2_) and stored at −80 °C awaiting processing. Cell density and average cell volume were measured from each replicate employing a Moxi^Z^ cell counter with type S cassettes (Orflo Technologies, Ketchum, ID, USA).

#### 3.2.2. Adherent Cell Lines

Five replicate cultures of the adherent cell lines Hek293 and HeLa S3 cells were seeded in 150 cm^2^ culture dishes (734–2322, VWR) and incubated for two days to reach ~80% confluency. Sampling was performed according to [39] with minor modifications. In brief, culture media was discarded before cells rapidly were rinsed in two consecutive steps, first with 20 mL cold saline, next with 20 mL cold MQ-H_2_O, both while placed on a cold (−80 °C) metal block. Next, cells were mechanically detached in 20 mL of cold MQ-H_2_O:ACN (1:1, *v/v*). The dish was rinsed with an additional 10 mL of MQ-H_2_O:ACN before cell suspension from both steps were collected into a centrifuge tube and quenched in LN_2_. The suspension was and stored at −80 °C awaiting processing. Cell density and average cell volume were measured from separate dishes, employing a Moxi^Z^ cell counter with type S cassettes (Orflo Technologies).

#### 3.2.3. Microorganisms 

*B. subtilis, S. cerevisiae, N.oceanica,* and *P.tricornutum* were sampled in exponential phase, the two former at OD_600_ 1. Five replicate cultures were sampled for all organisms and conditions. Sampling was performed according to the filtration protocol described for suspension cell lines, with the modifications listed in Table 1. Cells were harvested on Supor hydrophilic polyethersulfone filters with a pore size of 0.8 µm (60110, Pall, Port Washington, NY, USA). For determination of cell dry weight, an aliquot of cell suspension from each replicate was pelleted, washed (MQ-H2O, 4500× *g*), transferred to a pre-weighed aluminum pan, and left in an oven until constant weight was measured.

### 3.3. Preparation of Metabolite Extracts

Intracellular metabolites were extracted from quenched cell suspensions by cycling the MQ-H_2_O:ACN cell suspensions between LN_2_ and cold water (< 4 °C). Three repeated freeze-thaw cycles were carried out to allow for ice crystal formation and consequently, disruption of cell walls and/or membranes. Complete disruption for extraction of microalgae was performed by extending the protocol by three additional freeze-thaw cycles. Filters employed for harvesting suspension cells were removed before all extracts were cleared of cell debris by centrifugation (4 °C, 4500× *g*), concentrated by lyophilization, and stored at −80 °C. Upon analysis, lyophilized extracts were reconstituted in cold MQ-H_2_O, spin filtered (4 °C, 20,000× *g*) with 3 kDa molecular cutoff (516–0228P, VWR) and aliquoted for three tandem mass spectrometric (MS/MS) methods covering different metabolite classes.

### 3.4. Targeted Mass Spectrometric Metabolite Profiling

#### 3.4.1. CapIC-MS/MS Analysis of Phosphorylated Metabolites and TCA Cycle Intermediates

Phosphorylated metabolites and intermediates of the TCA cycle were quantified by capillary ion chromatography (capIC)-MS/MS. Metabolite extracts were processed and analyzed as described in [39] with the modifications described in [40], employing a Xevo TQ-XS triple quadrupole mass spectrometer (Waters, Milford, MA, USA).

#### 3.4.2. LC-MS/MS Analysis of Organic Acids

Metabolite extracts were added 20% (*v/v*) of an U^13^C-labeled *S. cerevisiae* extract prepared as described in [40] supplemented with U^13^C-pyruvic acid and –lactic acid (2440-0.5 and 1579-0.5, Cambridge isotope laboratories, Tewksbury, MA, USA), and derivatized as described in [70] to allow for quantification of organic acids. LC-MS/MS analysis was performed on an ACQUITY I-Class UPLC coupled to a Xevo TQ-XS triple quadrupole mass spectrometer (Waters) equipped with an electrospray source operating in positive mode. The capillary voltage was set to 3.0 V, the source and desolvation temperature to 150 °C and 620 °C, respectively, and the desolvation gas flow to 1000 L/h. Derivatized samples (5 μL) were injected onto a Waters Aquity BEH C18 2.1 × 100 mm column with a pore size of 1.7 µm (186002352, Waters) maintained at 40 °C and eluted with mobile phases (A) HPLC-grade water (83645.320, VWR) with 0.1% formic acid (*v/v*, 5.33002, Sigma-Aldrich) and (B) methanol (1.06035.2500, Merck Millipore The following gradient (*v/v*) was applied with a flow rate of 0.25 mL/min: 0–0.5 min; 50% B, 0.5–6 min: 50–99% B, 6–7 min: 99% B, 7–7.1 min: 100–50% B, 8 min: end. Precursor-product ion transitions were as described in [70].

#### 3.4.3. LC-MS/MS Analysis of Amino Acids

Metabolite extracts were added 5% (*v/v*) of a mixture containing 19 ^13^C, ^15^N-labeled amino acids (100 µM, MSK-A2-1.2, Cambridge Isotope Laboratories) and derivatized to allow for quantification of amino acids. In brief, extracts were concentrated under vacuum at 60° C, reconstituted in a mixture of ethanol (20824.365 VWR), HPLC-grade water (83645.320, VWR) and pyridine (270970, Sigma-Aldrich) (1:1:1, *v/v*), added 5% (*v/v*) of the derivatization reagent phenyl isothiocyanate (78780, Sigma-Aldrich). Next, the formation of phenylthiocarbamyl derivatives was allowed to take place at room temperature for 20 min, before derivatized extracts were concentrated under vacuum at 45 °C and reconstituted in methanol (1.06035.2500, Merck Millipore) with 5 mM ammonium acetate (73594, Sigma Aldrich). Derivatized samples were analyzed applying the UPLC-TQ-XS setup described above, with the electrospray source operating in positive mode. The capillary voltage was set to 3.2 V, the source and desolvation temperature to 150 °C and 500 °C, respectively, and the desolvation gas flow to 1000 L/h. Samples (2 µL) were injected onto an ACQUITY UPLC BEH C_18_ 2.1 × 75 mm column fitted with an ACQUITY UPLC BEH C_18_ 2.1 × 5 mm VanGuard pre-column, both with a pore size of 1.7 µm (186005604 and 186003975, Waters). The column was maintained at 50 °C and eluted with mobile phases (A) HPLC-grade water (83645.320, VWR) and (B) ACN (83640.320 VWR), both with 0.2% formic acid (*v/v*,5.33002, Sigma-Aldrich). The following gradient (*v/v*) was applied with a flow rate of 0.5 mL/min: 0–1: 0% B, 0.45–3.3 min: 0–15% B, 3.3–5.9 min: 15–70% B, 5.9–6.05 min: 70–100% B, 6.05–6.52 min: 100% B, 6.52–7.30 min: 0% B, 7.3 min: end. The 20 proteinogenic amino acids were quantified from precursor-product ion transitions listed in Table S6.

### 3.5. Data Analysis

#### 3.5.1. Data Processing

Data processing was performed in the TargetLynx application manager of MassLynx 4.1 (Waters). Absolute quantification was performed by interpolation from calibration curves prepared by serial dilutions of analytical grade standards (Sigma-Aldrich). Calibration curves were calculated by least-squares regression with 1/x weighting, and all response factors of both standards and biological extracts were corrected by the response factor of the corresponding U^13^C(^15^N)-isotopologue. Extract concentrations were corrected for dilutions and concentrations performed during sample preparation, and normalized to measured cell density (cells/L) or total DW (g/L) in cell lines and microorganisms, respectively. Intracellular metabolite concentrations were calculated from experimental cell volumes (pL) of cell lines and estimated using literature values for specific cell volume (L/g) of microorganisms (Table 2).

#### 3.5.2. Statistical Analysis

Outliers in the sub-sample sets were identified and rejected according to Dixon’s Q test [77]. Further statistical analyses were performed for metabolites present in > 50% of the panel in MetaboAnalyst 4.0 [49]. Missing values (4% of the total dataset) were estimated by the minimum measured value of the respective metabolite. Data were auto-scaled prior to PCA and two-tailed T-tests assuming equal variances (Adjusted *p*-value (FDR) = 0.05). Log_2_ fold changes of average metabolite levels were graphed to metabolic pathways in the Omix editor and modeling tool for metabolic network diagrams [78].

## Figures and Tables

**Table 1:** Sampling parameters employed for *Bacillus subtilis*, *Saccharomyces cerevisiae*, *Nannochloropsis oceanica,* and *Phaeodactylum tricornutum*, including vacuum pressure, sampling volume and filter type.

Organism	Vacuum Pressure (Below ambient, mbar)	Sampling Volume (mL)
*B. subtilis*	700	10
*S. cerevisiae*	400	10
*N. oceanica*	600	15
*P. tricornutum*	600	15

**Table 2:** Literature values listed for cell dry weight (DW), cell volume, and specific cell volume (L/g) employed to estimate intracellular concentration.

Organism	Cell DW (g/cell)	Cell Volume (L/cell)	Specific Cell Volume (L/g)	Reference
*B. subtilis*	2.2 × 10^−13^	9 × 10^−16^	4.09 × 10^−3^	[71]
*S. cerevisiae*	1.65 × 10^−11^	4.4 × 10^−14^	2.66 × 10^−3^	[72]
*N. oceanica*	1.19 × 10^−11^	1.4 × 10^−14^	1.18 × 10^−3^	* Cell volume [73], cell DW [74]
*P. tricornutum*	4.88 × 10^−11^	1.22 × 10^−13^	2.51 × 10^−3^	Cell volume [75], cell DW [76]

* *P. tricornutum cell* volume was calculated considering the half parallelepiped (length × width × height/2) shape of the organism, applying a width and height of 3.5 µm and a length of 20 µm.

## Supplementary Materials

The following are available online at https://www.mdpi.com/2218-1989/10/2/74/s1, Table S1: Metabolite abbreviations, Table S2: Intracellular concentrations, Table S3: Contribution of metabolite classes, Table S4: YMDB entries, Table S5: *Escherichia coli sampling parameters*, Table S6: Transitions of derivatized amino acids, Figure S1: PCA scores and loadings for human cell lines.
