# Supplementary material for: Absolute Quantification of the Central Carbon Metabolome in Eight Commonly Applied Prokaryotic and Eukaryotic Model Systems
Source: Metabolites. 2020 Feb 19;10(2):74. doi: 10.3390/metabo10020074 (PMC7073941; doi:10.3390/metabo10020074)
Supplement: Supplementary file 1 [file metabolites-10-00074-s001.zip › TableS6 Rost et al Feb2020.pdf]

Table S6 Transitions of derivatized amino acids: Retention time, collision potential, and precursor-product ion transition of $U^{12}C$ and $U^{13}C^{15}N$ isotopologues. Metabolite abbreviations are listed in Table S1.

Metabolite	Rentention time (min)	Collision (V)	^{12}C		$U^{13}C,^{15}N$	
			Q1	Q3	Q1	Q3
Ala	4,1	14	225,0	44,0	228,9	94,2
Arg	2,8	14	310,1	217,1	320,1	227,1
Asn	3,0	16	268,0	87,1	272,2	90,1
Asp	3,7	14	269,0	116,0	274,0	139,3
Cys	4,5	8	257,0	122,0	261,0	126,0
Gln	3,1	16	282,1	129,8	287,1	194,1
Glu	3,7	16	283,0	130,0	289,0	136,0
Gly	3,3	10	211,0	75,9	214,0	78,9
His	2,4	22	291,1	110,1	300,1	118,0
Ile	5,1	28	267,3	69,0	274,3	74,0
Leu	5,1	38	267,1	43,3	274,1	46,0
Lys	5,1	12	417,1	324,1	425,1	332,1
Met	4,8	18	285,1	104,0	291,1	108,0
Phe	5,1	26	301,1	136,0	311,1	136,0
Pro	4,1	24	251,0	136,0	257,0	136,0
Ser	3,2	16	241,1	60,0	245,1	63,0
Thr	4,0	18	255,0	74,0	259,0	77,0
Trp	5,0	28	340,1	146,0	-*	-*
Tyr	4,5	26	317,1	123,0	327,1	147,2
Val	4,8	18	253,0	136,0	259,0	136,0

*$U^{13}C,^{15}N$ Phe transition applied for correction
