# Supplementary material for: Absolute Quantification of the Central Carbon Metabolome in Eight Commonly Applied Prokaryotic and Eukaryotic Model Systems
Source: Metabolites. 2020 Feb 19;10(2):74. doi: 10.3390/metabo10020074 (PMC7073941; doi:10.3390/metabo10020074)
Supplement: Supplementary file 1 [file metabolites-10-00074-s001.zip › TableS1 Rost et al Jan2020.pdf]

Table S1 Metabolite abbreviations: Metabolites included in panel for metabolite profiling, sorted on metabolite class and listed with abbreviation, Yeast- and Human Metabolome Database (YMDB and HMDB) IDs, and CAS number. TCA; tricarboxylic acid, PPP; pentose phosphate pathway.

Class	Abbreviation	Metabolite	YMDB ID	HMDB ID	CAS number
Glycolysis	G6P	Glucose 6-phosphate	YMDB00947	HMDB0001401	56-73-5
	F6P	Fructose 6-phosphate	YMDB00078	HMDB0000124	643-13-0
	F1,6BP	Fructose 1,6-bisphosphate	YMDB00108	HMDB0001058	488-69-7
	2PG	2-Phosphoglyceric acid	YMDB00675	HMDB0000807	2553-59-5
	3PG	3-Phosphoglyceric acid	YMDB00756	HMDB0000362	820-11-1
	PEP	Phosphoenolpyruvic acid	YMDB00276	HMDB0000263	138-08-9
	Pyr	Pyruvic acid	YMDB00175	HMDB0000243	127-17-3
PPP	6PG	6-Phosphogluconic acid	YMDB00218	HMDB0001316	921-62-0
	R5P	Ribose 5-phosphate	YMDB00119	HMDB0001548	3615-55-2
	S7P	Sedoheptulose 7-phosphate	YMDB00752	HMDB0001068	2646-35-7
Other sugar phosphates	F1P	Fructose 1-phosphate	YMDB00655	HMDB0001076	15978-08-2
	G1P	Glucose 1-phosphate	YMDB00288	HMDB0001586	59-56-3
	M1P	Mannose 1-phosphate	YMDB00386	HMDB0006330	27251-84-9
	GAL1P	Galactose 1-phosphate	YMDB00004	HMDB0000645	2255-14-3
	GAL6P	Galactose 6-phosphate	YMDB02311	-	NA
	GL3P	Glycerol 3-phosphate	YMDB00073	HMDB0000126	17989-41-2
	M6P	Mannose 6-phosphate	YMDB00242	HMDB0001078	3672-15-9
	UDP-glu	Uridine diphosphate glucose	YMDB00415	HMDB0000286	133-89-1
	UDP-Glc-Nac	Uridine diphosphate-N-acetylglucosamine	YMDB00277	HMDB0000290	528-04-1
	PRPP	Phosphoribosyl pyrophosphate	YMDB00402	HMDB0000280	7540-64-9
	Lac	Lactic acid	YMDB00247	HMDB0000190	79-33-4
TCA cycle	Cit	Citric acid	YMDB00086	HMDB0000094	77-92-9
	ICit	Isocitric acid	YMDB00026	HMDB0000193	320-77-4
	aKG	a-Ketoglutaric acid	YMDB00153	HMDB0000208	328-50-7
	Suc	Succinic acid	YMDB00338	HMDB0000254	110-15-6
	Fum	Fumaric acid	YMDB00101	HMDB0000134	110-17-8
	Mal	Malic acid	YMDB00280	HMDB0000156	97-67-6
Nucleoside phosphates	AMP	Adenosine monophosphate	YMDB00097	HMDB0000045	61-19-8
	ADP	Adenosine diphosphate	YMDB00914	HMDB0001341	58-64-0
	ATP	Adenosine triphosphate	YMDB00109	HMDB0000538	56-65-5
	GMP	Guanosine monophosphate	YMDB00261	HMDB0001397	85-32-5
	GDP	Guanosine diphosphate	YMDB00257	HMDB0001201	146-91-8
	GTP	Guanosine triphosphate	YMDB00558	HMDB0001273	86-01-1
	CMP	Cytidine monophosphate	YMDB00406	HMDB0000095	63-37-6
	CDP	Cytidine diphosphate	YMDB00064	HMDB0001546	63-38-7
	CTP	Cytidine triphosphate	YMDB00279	HMDB0000082	65-47-4
	UMP	Uridine monophosphate	YMDB00049	HMDB0000288	58-97-9
	UDP	Uridine diphosphate	YMDB00307	HMDB0000295	58-98-0
	UTP	Uridine triphosphate	YMDB00326	HMDB0000285	63-39-8
Deoxynucleotide sugars	dAMP	Deoxyadenosine monophosphate	YMDB00451	HMDB0000905	653-63-4
	dADP	Deoxyadenosine diphosphate	YMDB00901	HMDB0001508	2793-06-8
	dATP	Deoxyadenosine triphosphate	YMDB00700	HMDB0001532	1927-31-7
	dGTP	Deoxyguanosine triphosphate	YMDB00744	HMDB0001440	2564-35-4
	dCTP	Deoxycytosine triphosphate	YMDB00658	HMDB0000998	2056-98-6
	dUMP	Deoxyuridine monophosphate	YMDB00221	HMDB0001409	964-26-1
	dTMP	Deoxythymidine monophosphate	YMDB00162	HMDB0001227	365-07-1
	dTDP	Deoxythymidine diphosphate	YMDB00205	HMDB0001274	491-97-4
	dTTP	Deoxythymidine triphosphate	YMDB00745	HMDB0001342	365-08-2
Amino acids	Ala	Alanine	YMDB00154	HMDB0000161	56-41-7
	Arg	Arginine	YMDB00592	HMDB0000517	74-79-3
	Asn	Asparagine	YMDB00226	HMDB0000168	70-47-3
	Asp	Aspartic acid	YMDB00896	HMDB0000191	56-84-8
	Cys	Cysteine	YMDB00046	HMDB0000574	52-90-4
	Gln	Glutamine	YMDB00002	HMDB0000641	56-85-9
	Glu	Glutamic acid	YMDB00271	HMDB0000148	56-86-0
	Gly	Glycine	YMDB00016	HMDB0000123	56-40-6
	His	Histidine	YMDB00369	HMDB0000177	71-00-1
	Ile	Isoleucine	YMDB00038	HMDB0000172	73-32-5
	Leu	Leucine	YMDB00387	HMDB0000687	61-90-5
	Lys	Lysine	YMDB00330	HMDB0000182	56-87-1
	Met	Methionine	YMDB00318	HMDB0000696	63-68-3
	Phe	Phenylalanine	YMDB00304	HMDB0000159	63-91-2
	Pro	Proline	YMDB00378	HMDB0000162	147-85-3
	Ser	Serine	YMDB00112	HMDB0000187	56-45-1
	Thr	Threonine	YMDB00214	HMDB0000167	72-19-5
	Trp	Tryptophan	YMDB00126	HMDB0000929	73-22-3
	Tyr	Tyrosine	YMDB00364	HMDB0000158	60-18-4
	Val	Valine	YMDB00152	HMDB0000883	72-18-4
