# Supplementary material for: Absolute Quantification of the Central Carbon Metabolome in Eight Commonly Applied Prokaryotic and Eukaryotic Model Systems
Source: Metabolites. 2020 Feb 19;10(2):74. doi: 10.3390/metabo10020074 (PMC7073941; doi:10.3390/metabo10020074)
Supplement: Supplementary file 1 [file metabolites-10-00074-s001.zip › TableS2 Rost et al Jan2020.pdf]

Table S2 Intracellular concentrations: Intracellular concentrations (M) of all metabolites inculded in the panel for metabolite profiling, listed for all biological replicates. LOD; Limit of detection, LOQ; limit of quantification. Metabolite abbreviations are listed in Table S1.

Organism/ cell line	Culture medium	Replica	Intracellular concentration (M)																					
			2-/3PG	6PG	ADP	aKG	Ala	AMP	Arg	Asn	Asp	ATP	CDP	Cit	CMP	CTP	Cys	dADP	dAMP	dATP	dCTP	dGTP	dTDP	dTMP
Bacillus subtilis	Mineral	1	4.15E-04	6.52E-05	3.59E-04	1.50E-04	1.31E-04	1.90E-04	1.52E-05	3.51E-06	7.73E-05	5.18E-04	2.24E-05	3.01E-04	3.98E-05	5.57E-05	<LOQ	4.18E-05	2.46E-06	2.69E-05	1.60E-05	7.73E-06	1.59E-05	5.97E-06
		2	3.81E-04	5.49E-05	3.85E-04	8.73E-05	9.81E-05	1.37E-04	1.35E-05	1.23E-06	9.01E-05	7.10E-04	2.69E-05	1.56E-04	4.80E-05	8.81E-05	<LOQ	3.91E-05	1.72E-06	3.18E-05	1.28E-05	7.67E-06	1.14E-05	5.34E-06
		3	3.36E-04	5.03E-05	3.24E-04	8.27E-05	6.86E-05	8.40E-05	7.84E-06	0.00E+00	5.68E-05	7.07E-04	2.64E-05	1.32E-04	2.72E-05	8.74E-05	<LOQ	3.84E-05	1.08E-06	3.25E-05	1.60E-05	1.03E-05	1.01E-05	3.92E-06
		4	2.17E-04	1.66E-05	3.75E-04	9.81E-05	1.15E-04	2.73E-04	4.36E-05	6.02E-06	1.72E-04	5.58E-04	3.23E-05	1.27E-04	4.21E-05	8.50E-05	<LOQ	4.76E-05	2.78E-06	3.00E-05	1.40E-05	1.04E-05	1.59E-05	4.51E-06
	Rich	1	2.19E-04	2.21E-05	2.80E-04	2.93E-05	2.64E-04	8.99E-05	1.37E-04	4.34E-06	1.51E-04	5.99E-04	1.37E-05	5.50E-05	1.98E-05	4.00E-05	<LOQ	5.27E-05	1.35E-06	4.35E-05	1.10E-05	1.36E-05	6.12E-06	1.90E-06
		2	2.98E-04	5.93E-06	4.32E-04	1.97E-05	2.45E-04	1.98E-04	9.78E-05	4.27E-06	1.18E-04	6.68E-04	1.43E-05	2.89E-05	1.75E-05	1.75E-05	<LOQ	7.74E-05	5.77E-06	4.29E-05	3.74E-06	9.46E-06	5.77E-06	3.95E-06
		3	1.75E-04	8.71E-06	3.87E-04	2.38E-05	3.17E-04	1.33E-04	1.32E-04	5.37E-06	1.56E-04	7.71E-04	7.85E-06	3.82E-05	1.08E-05	9.94E-06	<LOQ	6.49E-05	3.49E-06	4.82E-05	3.65E-06	8.12E-06	3.87E-06	2.26E-06
		4	2.20E-04	1.06E-05	3.29E-04	1.20E-05	1.31E-04	9.55E-05	7.31E-05	1.94E-06	6.10E-05	7.88E-04	7.35E-06	2.25E-05	9.34E-06	1.20E-05	<LOQ	6.82E-05	2.76E-06	5.51E-05	3.82E-06	1.14E-05	3.05E-06	2.23E-06
Saccaromyces cervisiae	Mineral	1	5.15E-04	1.73E-04	6.08E-04	9.83E-05	6.74E-03	1.60E-04	1.00E-02	9.65E-04	1.06E-02	1.34E-03	4.68E-05	5.65E-04	7.56E-06	2.39E-04	<LOQ	1.66E-05	2.77E-07	1.82E-05	6.82E-06	6.64E-06	4.06E-06	1.11E-06
		2	8.61E-04	2.72E-04	9.21E-04	1.43E-04	9.95E-03	2.54E-04	1.47E-02	1.50E-03	1.51E-02	1.91E-03	8.48E-05	9.09E-04	1.51E-05	3.54E-04	<LOQ	2.46E-05	4.92E-07	2.48E-05	8.98E-06	8.98E-06	7.01E-06	1.60E-06
		3	9.23E-04	2.68E-04	1.01E-03	1.29E-04	9.55E-03	2.38E-04	1.14E-02	1.23E-03	1.26E-02	2.22E-03	1.05E-04	7.47E-04	1.76E-05	3.99E-04	<LOQ	2.81E-05	5.53E-07	2.82E-05	1.21E-05	1.11E-05	9.07E-06	1.55E-06
		4	6.66E-04	1.73E-04	7.48E-04	1.26E-04	1.05E-02	2.06E-04	1.12E-02	1.17E-03	1.10E-02	1.69E-03	5.79E-05	6.22E-04	9.71E-06	3.07E-04	<LOQ	1.84E-05	4.92E-07	2.15E-05	8.48E-06	6.89E-06	4.67E-06	1.35E-06
		5	6.14E-04	1.51E-04	9.15E-04	9.06E-05	9.94E-03	2.55E-04	1.47E-02	1.39E-03	1.36E-02	1.81E-03	7.79E-05	9.91E-04	1.52E-05	3.25E-04	<LOQ	2.46E-05	4.43E-07	2.39E-05	9.41E-06	8.52E-06	7.97E-06	1.44E-06
	Rich	1	7.16E-04	7.28E-05	6.40E-04	6.67E-04	1.10E-02	2.47E-04	1.04E-02	2.10E-03	2.41E-02	1.27E-03	6.76E-05	7.96E-04	2.42E-05	2.82E-04	<LOQ	2.39E-05	8.30E-07	2.30E-05	9.04E-06	8.11E-06	8.39E-06	1.48E-06
		2	6.13E-04	6.98E-05	6.06E-04	5.11E-04	8.44E-03	2.81E-04	9.05E-03	1.44E-03	2.24E-02	1.03E-03	8.20E-05	7.03E-04	2.14E-05	2.21E-04	<LOQ	2.36E-05	1.03E-06	1.98E-05	7.43E-06	7.19E-06	9.01E-06	1.34E-06
		3	7.40E-04	7.79E-05	6.87E-04	7.62E-04	8.51E-03	2.21E-04	1.05E-02	1.53E-03	2.43E-02	1.22E-03	7.97E-05	8.48E-04	1.63E-05	2.69E-04	<LOQ	2.75E-05	9.36E-07	2.27E-05	8.51E-06	8.00E-06	9.02E-06	1.53E-06
		4	7.20E-04	8.19E-05	6.96E-04	6.59E-04	1.02E-02	2.67E-04	1.09E-02	1.92E-03	2.53E-02	1.21E-03	8.73E-05	7.53E-04	2.69E-05	2.64E-04	<LOQ	2.78E-05	1.20E-06	2.23E-05	8.11E-06	8.30E-06	1.10E-05	1.66E-06
Nannochloropsis oceanica	Mineral	1	8.10E-04	8.73E-06	4.02E-04	1.04E-03	3.91E-03	3.41E-04	3.78E-04	5.72E-04	6.83E-03	1.13E-03	1.84E-05	8.69E-03	2.13E-05	4.17E-05	<LOQ	4.85E-06	<LOQ	6.79E-06	1.94E-06	<LOQ	3.88E-06	3.88E-06
		2	8.98E-04	2.32E-05	6.05E-04	9.93E-04	5.51E-03	1.65E-04	6.36E-04	1.00E-03	8.40E-03	1.78E-03	1.76E-05	1.25E-02	1.30E-05	7.66E-05	<LOQ	3.25E-06	<LOQ	5.11E-06	1.86E-06	<LOQ	1.86E-06	2.32E-06
		3	5.48E-04	2.48E-06	4.75E-04	6.04E-04	3.35E-03	2.56E-04	4.34E-04	5.83E-04	4.96E-03	8.75E-04	2.11E-05	4.81E-03	2.11E-05	2.23E-05	<LOQ	2.48E-06	<LOQ	4.96E-06	1.24E-06	<LOQ	2.48E-06	4.96E-06
		4	1.29E-03	2.89E-05	6.24E-04	6.44E-04	3.06E-03	1.51E-04	3.80E-04	5.83E-04	5.47E-03	1.87E-03	1.87E-05	1.08E-02	2.03E-05	7.76E-05	<LOQ	4.28E-06	<LOQ	5.89E-06	2.14E-06	<LOQ	1.61E-06	3.21E-06
		5	8.53E-04	1.51E-05	4.58E-04	6.36E-04	3.67E-03	3.86E-04	5.12E-04	7.71E-04	6.28E-03	1.15E-03	2.45E-05	7.16E-03	2.02E-05	4.54E-05	<LOQ	2.88E-06	<LOQ	5.04E-06	1.44E-06	<LOQ	2.16E-06	2.16E-06
Phaeodactylum tricornotum	Mineral	1	2.10E-04	4.77E-06	3.28E-04	1.12E-04	2.78E-03	1.70E-04	1.93E-03	7.83E-03	3.70E-03	4.14E-04	2.86E-05	4.01E-03	6.50E-06	1.58E-05	<LOQ	2.38E-06	<LOD	<LOD	2.60E-06	<LOD	3.03E-06	2.82E-06
		2	2.13E-04	4.23E-06	2.07E-04	1.14E-04	2.54E-03	6.17E-05	1.65E-03	5.85E-03	2.69E-03	5.98E-04	9.47E-06	4.28E-03	4.23E-06	2.94E-05	<LOQ	6.05E-07	<LOD	<LOD	1.01E-06	<LOD	8.06E-07	1.41E-06
		3	3.08E-04	8.70E-06	2.40E-04	8.91E-03	2.78E-04	7.46E-05	4.89E-04	7.89E-04	4.60E-04	6.30E-04	8.31E-06	1.29E-02	4.45E-06	3.73E-05	<LOQ	7.73E-07	<LOD	<LOD	NA	<LOD	3.87E-07	5.80E-07
		4	2.29E-04	7.00E-06	2.32E-04	1.07E-02	2.38E-04	1.59E-04	2.48E-04	5.41E-04	4.48E-04	4.65E-04	1.63E-05	1.07E-02	8.07E-06	1.21E-05	<LOQ	6.37E-07	<LOD	<LOD	4.25E-07	<LOD	4.25E-07	1.49E-06
		5	3.01E-04	9.15E-06	2.46E-04	1.12E-02	2.66E-04	1.99E-04	2.82E-04	8.57E-04	5.21E-04	4.99E-04	1.77E-05	1.05E-02	1.05E-05	1.35E-05	<LOQ	5.96E-07	<LOD	<LOD	3.98E-07	<LOD	5.96E-07	1.59E-06
Hek293	Rich	1	9.15E-05	1.39E-05	8.25E-04	2.23E-04	3.94E-03	1.78E-04	1.21E-03	1.70E-04	1.84E-03	2.40E-03	9.02E-05	7.94E-04	1.22E-05	2.96E-04	<LOQ	1.29E-05	1.72E-07	1.32E-05	6.28E-06	3.27E-06	6.28E-06	1.03E-06
		2	7.58E-05	1.27E-05	8.39E-04	9.28E-04	3.48E-03	1.60E-04	9.56E-04	1.40E-04	1.63E-03	2.45E-03	9.02E-05	6.61E-04	1.27E-05	3.02E-04	<LOQ	1.32E-05	1.72E-07	1.40E-05	6.37E-06	3.36E-06	5.94E-06	9.47E-07
		3	7.24E-05	1.31E-05	7.75E-04	8.36E-04	2.87E-03	1.36E-04	8.45E-04	1.16E-04	1.43E-03	2.49E-03	8.03E-05	6.10E-04	1.02E-05	3.25E-04	<LOQ	1.22E-05	1.72E-07	1.39E-05	6.45E-06	3.61E-06	5.77E-06	9.47E-07
		4	7.87E-05	1.27E-05	7.99E-04	7.84E-04	2.89E-03	1.37E-04	9.17E-04	1.18E-04	1.39E-03	2.48E-03	8.00E-05	5.69E-04	1.08E-05	3.19E-04	<LOQ	1.21E-05	1.72E-07	1.27E-05	6.63E-06	3.53E-06	7.40E-06	9.47E-07
HeLa S3	Rich	1	4.40E-05	7.17E-05	6.61E-04	8.73E-05	4.68E-03	1.23E-04	1.33E-03	1.51E-04	2.00E-02	1.97E-03	4.94E-05	1.04E-03	6.05E-06	2.73E-04	<LOQ	1.01E-05	1.34E-07	1.18E-05	5.72E-06	3.16E-06	4.57E-06	1.08E-06
		2	5.82E-05	7.89E-05	6.75E-04	7.67E-05	4.20E-03	1.30E-04	1.23E-03	1.47E-04	1.68E-02	2.14E-03	5.14E-05	9.80E-04	5.85E-06	2.85E-04	<LOQ	9.82E-06	2.02E-07	1.21E-05	6.19E-06	3.50E-06	4.37E-06	1.14E-06
		3	9.21E-05	1.13E-04	9.23E-04	1.38E-04	5.23E-03	1.76E-04	1.36E-03	1.86E-04	2.67E-02	2.54E-03	9.07E-05	1.33E-03	1.87E-05	4.00E-04	<LOQ	1.57E-05	3.36E-07	1.67E-05	8.00E-06	4.91E-06	8.34E-06	1.08E-06
		4	5.10E-05	6.29E-05	7.16E-04	7.11E-05	4.67E-03	1.27E-04	1.22E-03	1.50E-04	1.97E-02	2.01E-03	5.18E-05	1.04E-03	5.65E-06	2.75E-04	<LOQ	1.03E-05	1.34E-07	1.17E-05	6.05E-06	2.96E-06	4.77E-06	1.01E-06
		5	7.28E-05	9.68E-05	7.78E-04	1.03E-04	4.08E-03	1.45E-04	1.49E-03	1.51E-04	1.68E-02	2.46E-03	5.96E-05	1.08E-03	7.06E-06	3.39E-04	<LOQ	1.22E-05	1.34E-07	1.40E-05	7.26E-06	3.70E-06	6.19E-06	1.21E-06
NB4	Rich	1	6.29E-05	8.66E-06	2.03E-04	9.97E-05	1.63E-03	6.90E-05	1.71E-03	1.08E-03	8.86E-04	5.62E-04	1.82E-05	2.40E-04	4.38E-06	7.08E-05	<LOQ	6.27E-06	<LOQ	7.05E-06	4.08E-06	3.40E-06	3.03E-06	3.15E-06
		2	1.12E-04	1.52E-05	3.99E-04	1.81E-04	2.89E-03	1.18E-04	2.68E-03	1.84E-03	1.65E-03	1.07E-03	2.69E-05	3.77E-04	5.26E-06	1.42E-04	<LOQ	9.19E-06	<LOQ	1.01E-05	4.11E-06	2.01E-06	3.70E-06	2.12E-06
		3	9.24E-05	1.37E-05	4.17E-04	1.50E-04	2.41E-03	1.23E-04	2.22E-03	1.56E-03	1.36E-03	1.03E-03	2.86E-05	3.46E-04	6.01E-06	1.31E-04	<LOQ	8.57E-06	<LOQ	9.54E-06	3.70E-06	1.78E-06	3.58E-06	1.91E-06
		4	5.73E-05	8.97E-06	2.50E-04	7.72E-05	1.50E-03	8.58E-05	1.37E-03	9.23E-04	8.29E-04	6.25E-04	1.98E-05	2.30E-04	4.67E-06	7.10E-05	<LOQ	5.04E-06	<LOQ	5.95E-06	2.25E-06	1.09E-06	2.22E-06	1.46E-06
MC/CAR	Rich	1	7.50E-05	5.29E-06	5.99E-04	1.93E-04	4.35E-03	1.84E-04	1.28E-03	1.00E-03	4.70E-03	1.39E-03	4.18E-05	5.33E-04	9.06E-06	1.34E-04	<LOQ	7.66E-06	<LOQ	9.79E-06	5.61E-06	1.80E-06	<LOQ	2.58E-06
		2	7.91E-05	5.74E-06	6.52E-04	2.06E-04	4.07E-03	1.98E-04	1.24E-03	9.85E-04	4.43E-03	1.46E-03	4.84E-05	6.04E-04	1.12E-05	1.45E-04	<LOQ	9.02E-06	<LOQ	1.03E-05	6.22E-06	1.71E-06	<LOQ	2.85E-06
		3	7.17E-05	3.93E-06	5.86E-04	1.96E-04	4.06E-03	1.84E-04	1.22E-03	9.47E-04	4.07E-03	1.35E-03	4.22E-05	5.72E-04	9.12E-06	1.26E-04	<LOQ	8.38E-06	<LOQ	9.42E-06	5.28E-06	1.61E-06	<LOQ	2.66E-06
		4	6.64E-05	3.54E-06	5.43E-04	2.07E-04	5.46E-03	1.75E-04	1.69E-03	1.27E-03	5.28E-03	1.27E-03	3.72E-05	6.21E-04	9.28E-06	1.14E-04	<LOQ	6.56E-06	<LOQ	9.10E-06	4.83E-06	1.38E-06	<LOQ	2.50E-06

Table S2 Intracellular concentrations: Continued

Organism/ cell line	Culture medium	Replica	Intracellular concentration (M)																					
			dTTP	dUMP	F1,6BP	F1P	F6P	Fum	G-/M1P	G6P	GAL1P	GAL6P	GDP	GL3P	Gln	Glu	Gly	GMP	GTP	His	Icit	Ile	Lac	Leu
Bacillus subtilis	Mineral	1	2.20E-05	7.02E-07	1.96E-04	4.10E-06	1.46E-04	4.80E-06	1.79E-05	1.51E-04	7.38E-06	1.17E-06	1.18E-04	2.93E-05	2.46E-05	1.72E-03	6.79E-05	2.70E-05	2.03E-04	8.20E-06	<LOQ	1.17E-05	8.50E-04	3.63E-05
		2	2.00E-05	4.29E-07	3.47E-04	4.48E-06	7.38E-05	3.07E-06	1.11E-05	7.19E-05	4.91E-06	6.13E-07	1.01E-04	3.04E-05	2.33E-05	2.13E-03	2.88E-05	1.61E-05	2.13E-04	3.07E-06	<LOQ	9.20E-06	1.85E-04	3.00E-05
		3	1.99E-05	4.90E-07	4.09E-04	3.72E-06	5.81E-05	1.96E-06	1.07E-05	5.97E-05	3.53E-06	1.18E-06	1.12E-04	2.22E-05	2.74E-05	1.44E-03	2.25E-05	1.81E-05	2.83E-04	2.94E-06	<LOQ	6.86E-06	2.65E-04	1.86E-05
		4	1.80E-05	6.77E-07	6.37E-04	1.26E-05	4.40E-05	2.63E-06	9.48E-06	4.28E-05	4.14E-06	3.01E-06	1.49E-04	1.84E-05	2.81E-04	2.94E-03	8.58E-05	3.71E-05	2.64E-04	1.58E-05	<LOQ	2.63E-05	7.61E-04	6.85E-05
	Rich	1	1.14E-05	1.08E-07	4.05E-04	3.85E-06	8.51E-05	4.06E-06	1.21E-05	7.56E-05	1.86E-04	8.13E-07	1.46E-04	1.51E-05	2.82E-05	9.42E-04	6.61E-05	2.37E-05	4.33E-04	2.44E-05	<LOQ	1.13E-04	1.38E-04	5.31E-04
		2	5.66E-06	2.67E-07	1.28E-03	9.62E-06	2.29E-05	6.95E-06	3.26E-06	1.38E-05	2.88E-05	2.46E-06	2.09E-04	6.79E-06	2.24E-05	4.67E-04	6.79E-05	8.45E-05	3.29E-04	2.19E-05	<LOQ	8.82E-05	3.45E-04	4.38E-04
		3	5.11E-06	1.07E-07	6.62E-04	5.48E-06	2.03E-05	6.23E-06	4.68E-06	1.91E-05	6.68E-05	1.02E-06	1.83E-04	8.33E-06	2.04E-05	6.20E-04	9.73E-05	5.04E-05	4.06E-04	3.12E-05	<LOQ	1.24E-04	4.31E-04	5.84E-04
		4	5.42E-06	9.68E-08	1.31E-03	7.74E-06	2.29E-05	4.16E-06	3.44E-06	1.79E-05	5.36E-05	1.69E-06	1.66E-04	6.39E-06	1.35E-05	3.52E-04	3.00E-05	4.35E-05	4.41E-04	1.06E-05	<LOQ	4.40E-05	1.42E-04	2.08E-04
Saccaromyces cervisiae	Mineral	1	1.27E-05	1.84E-07	3.39E-03	3.98E-05	4.68E-05	5.68E-05	7.56E-06	3.85E-05	1.12E-05	<LOD	1.16E-04	9.75E-05	2.93E-02	4.72E-02	1.72E-03	3.20E-05	3.40E-04	1.43E-03	7.19E-06	4.37E-04	1.40E-03	4.70E-04
		2	1.92E-05	2.46E-07	5.43E-03	5.72E-05	8.86E-05	7.43E-05	1.51E-05	7.60E-05	2.09E-05	<LOD	1.84E-04	1.37E-04	4.16E-02	7.10E-02	2.31E-03	5.48E-05	4.82E-04	2.02E-03	1.22E-05	6.04E-04	1.12E-03	6.32E-04
		3	2.05E-05	2.21E-07	6.75E-03	5.92E-05	8.61E-05	7.64E-05	1.34E-05	7.14E-05	2.18E-05	<LOD	2.01E-04	1.20E-04	3.71E-02	6.30E-02	2.07E-03	6.00E-05	5.87E-04	1.70E-03	1.42E-05	5.49E-04	9.72E-04	6.30E-04
		4	1.57E-05	1.23E-07	3.54E-03	4.13E-05	6.73E-05	6.76E-05	1.39E-05	5.85E-05	1.75E-05	<LOD	1.51E-04	1.21E-04	3.80E-02	6.01E-02	1.91E-03	4.48E-05	4.28E-04	1.66E-03	7.50E-06	5.27E-04	1.09E-03	6.10E-04
		5	1.77E-05	2.21E-07	3.17E-03	4.23E-05	6.31E-05	7.08E-05	9.41E-06	5.62E-05	1.83E-05	<LOD	1.79E-04	1.30E-04	4.04E-02	6.53E-02	2.19E-03	5.12E-05	4.91E-04	1.91E-03	1.21E-05	5.71E-04	6.74E-04	6.34E-04
	Rich	1	1.66E-05	<LOQ	3.94E-03	7.88E-05	8.96E-05	7.61E-05	1.25E-05	7.88E-05	2.98E-05	<LOD	1.62E-04	7.42E-05	5.16E-03	6.41E-02	3.55E-03	7.01E-05	4.35E-04	3.82E-03	4.16E-05	2.88E-03	7.86E-04	1.50E-02
		2	1.36E-05	<LOQ	3.11E-03	4.84E-05	8.25E-05	7.16E-05	1.15E-05	6.85E-05	2.21E-05	<LOD	1.68E-04	6.66E-05	5.14E-03	6.04E-02	2.85E-03	7.25E-05	3.77E-04	3.66E-03	3.00E-05	1.55E-03	5.70E-04	8.39E-03
		3	1.57E-05	<LOQ	3.77E-03	6.29E-05	9.93E-05	7.58E-05	1.39E-05	8.43E-05	2.76E-05	<LOD	2.14E-04	8.72E-05	5.97E-03	6.57E-02	2.92E-03	7.10E-05	4.43E-04	4.03E-03	4.57E-05	1.47E-03	6.21E-04	7.73E-03
		4	1.73E-05	<LOQ	4.02E-03	7.28E-05	1.10E-04	7.55E-05	1.49E-05	9.79E-05	2.66E-05	<LOD	2.00E-04	7.00E-05	5.58E-03	6.82E-02	3.53E-03	8.80E-05	4.25E-04	4.16E-03	3.75E-05	2.39E-03	7.21E-04	1.26E-02
Nannochloropsis oceanica	Mineral	1	<LOQ	<LOQ	9.31E-05	1.26E-05	5.23E-04	2.23E-05	9.02E-05	5.92E-04	1.13E-04	4.85E-06	4.56E-05	4.27E-05	2.96E-02	5.40E-02	1.89E-03	1.77E-04	1.14E-04	1.07E-04	2.33E-05	2.04E-04	1.52E-03	3.59E-04
		2	<LOQ	<LOQ	1.79E-04	2.14E-05	4.31E-04	1.49E-05	7.43E-05	4.85E-04	3.81E-05	3.71E-06	6.82E-05	6.22E-05	3.95E-02	6.53E-02	1.70E-03	1.27E-04	2.09E-04	1.49E-04	3.11E-05	2.79E-04	2.18E-03	4.64E-04
		3	<LOQ	<LOQ	4.47E-05	1.49E-05	3.89E-04	2.48E-05	6.70E-05	4.75E-04	9.55E-05	4.96E-06	3.47E-05	5.71E-05	2.56E-02	4.18E-02	1.56E-03	1.60E-04	6.08E-05	1.24E-04	1.61E-05	1.86E-04	2.65E-03	2.85E-04
		4	<LOQ	<LOQ	5.56E-05	2.46E-05	4.26E-04	1.77E-05	9.68E-05	4.90E-04	5.30E-05	3.75E-06	6.37E-05	6.47E-05	2.60E-02	4.43E-02	1.52E-03	1.34E-04	1.97E-04	1.07E-04	3.10E-05	2.57E-04	1.57E-03	4.55E-04
		5	<LOQ	<LOQ	2.09E-05	1.30E-05	4.14E-04	1.51E-05	8.43E-05	4.71E-04	5.40E-05	3.60E-06	4.68E-05	4.32E-05	3.19E-02	5.21E-02	2.05E-03	1.04E-04	1.28E-04	1.44E-04	1.95E-05	3.17E-04	3.56E-03	4.68E-04
Phaeodactylum tricornotum	Mineral	1	<LOQ	<LOQ	7.06E-05	3.25E-06	2.74E-04	9.53E-06	4.38E-05	2.93E-04	4.33E-05	2.17E-06	5.74E-05	2.60E-05	2.00E-02	2.19E-02	5.76E-04	4.98E-05	6.96E-05	2.38E-05	1.08E-05	9.10E-05	8.20E-04	1.13E-04
		2	<LOQ	<LOQ	6.13E-05	3.22E-06	2.72E-04	2.56E-05	4.55E-05	3.25E-04	3.41E-05	1.41E-06	3.10E-05	2.78E-05	1.44E-02	1.59E-02	7.17E-04	2.58E-05	9.39E-05	7.86E-05	5.64E-06	1.81E-04	2.34E-03	4.47E-04
		3	<LOQ	<LOQ	5.90E-05	2.90E-06	3.91E-04	1.64E-04	7.00E-05	4.40E-04	5.93E-05	2.13E-06	2.71E-05	4.74E-05	7.69E-04	8.93E-03	2.03E-04	4.48E-05	7.25E-05	1.93E-05	1.86E-05	8.70E-05	7.76E-04	1.55E-04
		4	<LOQ	<LOQ	3.63E-05	2.76E-06	3.34E-04	5.35E-05	6.69E-05	3.85E-04	4.46E-05	1.49E-06	2.23E-05	3.99E-05	4.39E-04	1.03E-02	2.14E-04	9.85E-05	4.52E-05	2.55E-05	1.02E-05	1.04E-04	5.82E-04	2.00E-04
		5	<LOQ	<LOQ	4.49E-05	2.98E-06	3.97E-04	3.72E-05	6.90E-05	4.37E-04	5.17E-05	1.79E-06	2.37E-05	5.43E-05	8.03E-04	1.28E-02	2.29E-04	1.12E-04	4.81E-05	1.99E-05	1.67E-05	9.15E-05	6.38E-04	1.71E-04
Hek293	Rich	1	1.29E-05	2.58E-07	1.60E-05	9.20E-05	5.58E-05	1.83E-04	1.98E-05	5.26E-05	1.19E-05	2.19E-05	1.59E-04	3.10E-04	9.14E-02	6.02E-02	8.24E-03	2.20E-05	5.23E-04	9.38E-04	1.86E-05	3.09E-03	4.37E-02	5.82E-03
		2	1.32E-05	2.58E-07	1.44E-05	1.04E-04	5.48E-05	1.72E-04	2.04E-05	5.19E-05	1.02E-05	1.87E-05	1.63E-04	3.40E-04	8.43E-02	5.51E-02	7.73E-03	2.27E-05	5.43E-04	7.34E-04	1.57E-05	2.29E-03	3.94E-02	4.44E-03
		3	1.39E-05	1.72E-07	NA	9.17E-05	5.29E-05	1.46E-04	1.87E-05	4.74E-05	1.09E-05	1.91E-05	1.51E-04	3.16E-04	7.49E-02	4.91E-02	6.76E-03	2.22E-05	5.45E-04	6.68E-04	1.64E-05	2.10E-03	3.06E-02	4.39E-03
		4	1.41E-05	1.72E-07	1.19E-05	8.40E-05	5.80E-05	1.30E-04	1.82E-05	5.34E-05	9.29E-06	2.15E-05	1.51E-04	3.31E-04	7.69E-02	4.89E-02	6.99E-03	2.35E-05	5.47E-04	6.90E-04	1.52E-05	2.23E-03	2.84E-02	4.38E-03
HeLa S3	Rich	1	1.02E-05	1.34E-07	1.39E-05	2.43E-05	1.19E-04	2.86E-04	3.99E-05	1.28E-04	1.45E-05	5.11E-06	1.01E-04	1.10E-04	7.66E-02	3.61E-02	1.02E-02	1.50E-05	4.03E-04	1.35E-03	2.08E-05	4.50E-03	3.42E-02	8.50E-03
		2	1.05E-05	1.34E-07	1.04E-05	2.49E-05	1.11E-04	2.57E-04	4.20E-05	1.09E-04	1.67E-05	4.64E-06	1.00E-04	1.16E-04	7.04E-02	3.27E-02	9.75E-03	1.34E-05	4.47E-04	1.28E-03	2.22E-05	4.10E-03	3.12E-02	7.64E-03
		3	1.51E-05	2.02E-07	5.99E-04	NA	1.48E-04	3.29E-04	5.26E-05	1.48E-04	1.94E-05	7.46E-06	1.49E-04	1.48E-04	7.83E-02	4.33E-02	1.04E-02	5.22E-05	6.09E-04	1.39E-03	3.13E-05	4.74E-03	3.02E-02	8.69E-03
		4	1.06E-05	1.34E-07	6.86E-06	2.53E-05	1.02E-04	2.79E-04	3.48E-05	9.76E-05	1.32E-05	5.04E-06	1.04E-04	1.11E-04	7.59E-02	3.63E-02	1.02E-02	1.51E-05	4.20E-04	1.34E-03	1.94E-05	4.52E-03	2.99E-02	8.36E-03
		5	1.26E-05	1.34E-07	NA	2.16E-05	1.42E-04	2.80E-04	5.02E-05	1.50E-04	2.29E-05	8.14E-06	1.16E-04	1.38E-04	7.20E-02	3.30E-02	1.04E-02	1.95E-05	5.11E-04	1.33E-03	2.60E-05	4.56E-03	4.07E-02	8.39E-03
NB4	Rich	1	7.48E-06	<LOQ	6.75E-05	1.89E-05	3.87E-05	2.23E-04	6.74E-06	2.82E-05	2.48E-06	<LOD	4.28E-05	2.00E-04	7.62E-03	4.94E-03	7.76E-04	1.14E-05	1.43E-04	1.91E-04	<LOQ	6.25E-04	1.22E-02	6.07E-04
		2	9.23E-06	<LOQ	3.92E-05	4.68E-05	8.14E-05	2.66E-04	1.39E-05	6.49E-05	6.20E-06	<LOD	6.96E-05	3.52E-04	1.31E-02	8.63E-03	1.35E-03	1.22E-05	2.86E-04	3.10E-04	<LOQ	1.04E-03	2.32E-02	1.02E-03
		3	9.03E-06	<LOQ	3.10E-05	3.60E-05	7.60E-05	4.57E-04	1.30E-05	5.92E-05	5.35E-06	<LOD	7.73E-05	3.27E-04	1.08E-02	7.13E-03	1.14E-03	1.16E-05	2.68E-04	2.54E-04	<LOQ	8.79E-04	1.87E-02	8.12E-04
		4	4.67E-06	<LOQ	7.82E-05	2.00E-05	4.10E-05	2.75E-04	7.72E-06	3.56E-05	3.58E-06	<LOD	4.82E-05	2.04E-04	6.49E-03	4.75E-03	6.47E-04	1.11E-05	1.62E-04	1.56E-04	<LOQ	5.22E-04	8.93E-03	4.98E-04
MC/CAR	Rich	1	1.08E-05	<LOQ	1.68E-05	4.96E-05	4.50E-05	6.68E-04	7.33E-06	3.98E-05	3.11E-06	<LOD	9.91E-05	4.63E-05	2.30E-02	2.04E-02	3.88E-03	2.22E-05	3.55E-04	9.22E-04	<LOD	3.07E-03	2.05E-02	3.16E-03
		2	9.81E-06	<LOQ	3.38E-05	6.05E-05	5.13E-05	9.68E-04	8.50E-06	4.24E-05	3.50E-06	<LOD	1.10E-04	5.93E-05	2.30E-02	1.93E-02	3.57E-03	2.42E-05	3.71E-04	8.15E-04	<LOD	3.01E-03	2.03E-02	2.89E-03
		3	7.68E-06	<LOQ	1.29E-05	5.07E-05	4.19E-05	8.16E-04	6.98E-06	3.39E-05	2.75E-06	<LOD	1.02E-04	5.23E-05	2.25E-02	1.81E-02	3.16E-03	2.20E-05	3.41E-04	7.94E-04	<LOD	2.93E-03	1.83E-02	2.89E-03
		4	7.59E-06	<LOQ	1.01E-05	4.72E-05	6.11E-05	6.77E-04	7.21E-06	5.38E-05	1.77E-06	<LOD	9.26E-05	4.62E-05	2.94E-02	2.18E-02	4.38E-03	1.95E-05	3.12E-04	1.10E-03	<LOD	4.11E-03	2.25E-02	3.92E-03

Table S2 Intracellular concentrations: Continued

Organism/ cell line	Culture medium	Replica	Intracellular concentration (M)																					
			**Lys**	**M6P**	**Mal**	**Met**	**PEP**	**Phe**	**Pro**	**PRPP**	**Pyr**	**R5P**	**S7P**	**Ser**	**Suc**	**Thr**	**Trp**	**Tyr**	**UDP**	**UDP-Glc-Nac**	**UDP-glu**	**UMP**	**UTP**	**Val**
Bacillus subtilis	Mineral	1	2.22E-05	4.17E-05	4.80E-06	1.17E-05	4.47E-05	5.85E-06	8.20E-06	4.77E-05	1.16E-04	7.17E-05	2.33E-04	8.43E-05	8.37E-05	2.93E-05	<LOQ	1.05E-05	3.42E-05	1.29E-04	8.32E-05	7.26E-05	1.46E-04	3.16E-05
		2	1.84E-05	2.99E-05	3.62E-06	7.97E-06	3.89E-05	5.52E-06	4.29E-06	1.43E-04	3.69E-05	9.85E-05	1.95E-04	3.80E-05	4.71E-05	2.39E-05	<LOQ	7.97E-06	2.64E-05	1.17E-04	1.01E-04	8.09E-05	1.66E-04	2.70E-05
		3	4.90E-06	2.76E-05	3.04E-06	6.86E-06	2.52E-05	2.94E-06	4.90E-06	1.10E-04	3.05E-05	7.35E-05	1.31E-04	3.04E-05	6.23E-05	1.18E-05	<LOQ	3.92E-06	9.21E-06	1.35E-04	8.65E-05	6.89E-05	1.60E-04	1.86E-05
		4	4.06E-05	2.43E-05	5.49E-06	1.13E-05	7.37E-06	1.66E-05	1.58E-05	9.58E-05	8.20E-05	7.13E-05	1.22E-04	7.83E-05	6.70E-05	5.27E-05	<LOQ	1.66E-05	2.53E-05	1.63E-04	1.11E-04	8.56E-05	1.44E-04	4.36E-05
	Rich	1	2.93E-04	3.24E-05	1.02E-05	4.93E-05	1.54E-05	1.21E-04	3.36E-05	1.74E-04	6.05E-05	6.54E-05	5.79E-05	8.56E-05	3.61E-05	9.48E-05	8.67E-06	2.49E-05	1.32E-05	9.20E-05	3.08E-05	2.67E-05	5.52E-05	1.41E-04
		2	2.06E-04	8.34E-06	1.41E-05	4.06E-05	2.77E-05	9.67E-05	2.78E-05	2.44E-05	3.45E-05	2.49E-05	3.71E-05	8.44E-05	4.19E-05	8.39E-05	4.27E-06	2.08E-05	1.77E-05	6.01E-05	1.86E-05	2.17E-05	4.03E-05	1.14E-04
		3	2.83E-04	1.03E-05	1.13E-05	5.43E-05	1.32E-05	1.31E-04	3.76E-05	1.42E-05	4.90E-05	2.84E-05	2.71E-05	1.26E-04	3.97E-05	1.09E-04	1.34E-05	2.90E-05	9.84E-06	5.27E-05	1.83E-05	1.64E-05	2.67E-05	1.55E-04
		4	1.41E-04	8.81E-06	1.16E-05	1.89E-05	1.35E-05	4.69E-05	1.40E-05	2.59E-05	2.08E-05	2.94E-05	3.40E-05	3.82E-05	3.41E-05	4.21E-05	1.45E-06	1.02E-05	7.89E-06	5.04E-05	1.37E-05	1.17E-05	2.50E-05	5.71E-05
Saccaromyces cervisiae	Mineral	1	2.97E-03	2.35E-05	2.56E-04	1.14E-04	4.44E-05	3.14E-04	5.82E-04	5.95E-05	1.52E-03	1.02E-04	2.98E-04	2.64E-03	3.51E-04	2.87E-03	5.44E-05	2.34E-04	5.65E-05	2.82E-04	2.63E-04	1.27E-04	2.99E-04	1.58E-03
		2	4.36E-03	4.80E-05	3.68E-04	1.57E-04	7.52E-05	4.05E-04	8.29E-04	8.31E-05	1.89E-03	1.29E-04	4.58E-04	3.80E-03	5.28E-04	3.82E-03	7.13E-05	3.18E-04	9.68E-05	4.16E-04	4.34E-04	1.97E-04	4.35E-04	2.29E-03
		3	3.45E-03	5.17E-05	3.85E-04	1.49E-04	7.98E-05	3.95E-04	7.41E-04	1.50E-04	1.67E-03	1.58E-04	4.97E-04	3.35E-03	5.81E-04	3.12E-03	6.64E-05	3.16E-04	1.23E-04	4.81E-04	4.44E-04	2.09E-04	5.16E-04	2.08E-03
		4	2.95E-03	3.59E-05	3.04E-04	1.40E-04	6.47E-05	3.86E-04	7.24E-04	6.22E-05	1.56E-03	1.50E-04	3.37E-04	3.06E-03	4.93E-04	2.98E-03	6.52E-05	3.11E-04	6.93E-05	3.75E-04	3.85E-04	1.75E-04	3.99E-04	2.22E-03
		5	4.22E-03	2.88E-05	3.42E-04	1.44E-04	6.20E-05	3.92E-04	7.69E-04	7.98E-05	1.48E-03	1.16E-04	3.67E-04	3.53E-03	5.15E-04	3.48E-03	6.97E-05	3.16E-04	9.37E-05	4.09E-04	3.50E-04	1.68E-04	4.28E-04	2.14E-03
	Rich	1	1.31E-02	4.99E-05	4.03E-04	1.66E-03	8.51E-05	3.32E-03	1.91E-03	7.13E-05	1.66E-03	3.70E-05	1.18E-04	4.88E-03	6.49E-04	4.99E-03	7.35E-04	7.14E-04	1.14E-04	3.84E-04	3.73E-04	1.92E-04	4.68E-04	3.99E-03
		2	9.52E-03	4.62E-05	3.71E-04	1.01E-03	8.09E-05	1.86E-03	1.57E-03	5.77E-05	1.31E-03	3.07E-05	1.08E-04	3.87E-03	4.85E-04	3.96E-03	3.14E-04	3.90E-04	1.27E-04	3.82E-04	3.79E-04	1.74E-04	3.77E-04	2.43E-03
		3	1.09E-02	4.70E-05	3.94E-04	1.01E-03	8.78E-05	1.72E-03	1.58E-03	7.02E-05	1.73E-03	3.78E-05	1.25E-04	3.95E-03	5.22E-04	4.44E-03	2.58E-04	3.72E-04	1.34E-04	4.56E-04	4.40E-04	2.02E-04	4.54E-04	2.29E-03
		4	1.24E-02	5.53E-05	3.86E-04	1.44E-03	8.63E-05	2.81E-03	1.86E-03	7.52E-05	1.38E-03	3.30E-05	1.21E-04	4.78E-03	5.69E-04	4.89E-03	5.63E-04	6.39E-04	1.42E-04	4.21E-04	3.96E-04	2.01E-04	4.54E-04	3.45E-03
Nannochloropsis oceanica	Mineral	1	1.59E-03	3.39E-04	9.41E-05	1.55E-04	9.70E-05	2.91E-04	1.38E-01	1.28E-04	8.76E-04	4.07E-05	4.16E-04	1.64E-03	6.39E-04	6.69E-04	5.82E-05	2.43E-04	2.52E-05	4.75E-05	1.86E-04	1.86E-04	8.63E-05	8.92E-04
		2	1.69E-03	2.26E-04	1.12E-04	2.14E-04	7.43E-05	3.06E-04	1.58E-01	2.18E-05	4.02E-04	3.53E-05	3.09E-04	1.81E-03	2.64E-04	8.08E-04	5.57E-05	2.27E-04	2.88E-05	5.76E-05	2.01E-04	2.01E-04	1.48E-04	1.22E-03
		3	1.03E-03	1.98E-04	1.07E-04	2.48E-05	6.82E-05	2.36E-04	1.25E-01	1.49E-05	1.11E-03	3.35E-05	3.50E-04	1.13E-03	5.37E-04	5.46E-04	2.48E-05	1.49E-04	2.98E-05	4.59E-05	2.07E-04	2.01E-04	4.59E-05	6.95E-04
		4	1.11E-03	2.80E-04	1.25E-04	1.34E-04	1.44E-04	2.89E-04	1.20E-01	7.49E-06	3.87E-04	2.14E-05	3.00E-04	1.46E-03	2.96E-04	5.51E-04	3.75E-05	1.77E-04	3.48E-05	5.99E-05	1.80E-04	2.19E-04	1.73E-04	9.42E-04
		5	1.43E-03	2.66E-04	8.29E-05	1.15E-04	1.15E-04	3.60E-04	1.49E-01	5.04E-06	3.41E-04	2.67E-05	3.59E-04	1.74E-03	3.86E-04	7.57E-04	4.32E-05	2.16E-04	3.89E-05	3.89E-05	1.28E-04	1.51E-04	1.07E-04	1.07E-03
Phaeodactylum tricornotum	Mineral	1	2.69E-03	6.89E-05	5.70E-05	<LOD	1.26E-05	8.45E-05	3.63E-02	1.32E-05	2.60E-05	3.14E-05	2.74E-04	4.16E-04	1.29E-04	2.77E-04	2.17E-06	2.38E-05	2.77E-05	8.23E-06	5.85E-05	7.06E-05	2.58E-05	4.55E-04
		2	2.16E-03	1.02E-04	5.91E-05	<LOD	1.53E-05	1.51E-04	3.26E-02	1.73E-05	8.46E-05	3.33E-05	4.75E-04	5.76E-04	1.06E-04	3.53E-04	1.41E-05	6.25E-05	8.67E-06	8.46E-06	5.62E-05	7.09E-05	4.55E-05	5.28E-04
		3	3.46E-04	1.67E-04	2.61E-04	<LOD	3.13E-05	5.22E-05	2.09E-02	7.35E-06	7.11E-05	4.89E-05	7.46E-04	3.87E-04	9.61E-04	1.68E-04	2.13E-05	4.06E-05	5.41E-06	1.66E-05	7.89E-05	1.18E-04	2.86E-05	3.13E-04
		4	2.84E-04	1.80E-04	1.34E-04	<LOD	1.95E-05	5.52E-05	1.66E-02	8.28E-06	6.39E-05	4.59E-05	7.87E-04	4.42E-04	9.00E-04	1.51E-04	2.76E-05	4.88E-05	8.92E-06	9.55E-06	5.56E-05	9.57E-05	1.25E-05	3.18E-04
		5	2.80E-04	2.15E-04	1.72E-04	<LOD	2.35E-05	6.16E-05	2.32E-02	5.96E-06	8.49E-05	4.69E-05	8.38E-04	4.23E-04	1.32E-03	1.57E-04	2.19E-05	3.98E-05	9.74E-06	1.39E-05	7.77E-05	1.37E-04	1.47E-05	3.10E-04
Hek293	Rich	1	3.35E-03	4.17E-05	7.17E-04	1.01E-03	5.51E-06	1.86E-03	3.43E-03	2.07E-05	1.01E-03	2.12E-05	2.51E-05	2.12E-03	3.01E-04	7.71E-03	4.19E-04	1.81E-03	1.57E-04	6.22E-04	2.36E-04	1.66E-04	6.23E-04	3.24E-03
		2	2.59E-03	4.01E-05	6.53E-04	8.50E-04	5.42E-06	1.43E-03	3.40E-03	2.25E-05	5.87E-04	2.03E-05	2.63E-05	1.71E-03	2.22E-04	7.01E-03	3.58E-04	1.40E-03	1.61E-04	6.51E-04	2.54E-04	1.67E-04	6.49E-04	2.45E-03
		3	2.36E-03	3.45E-05	5.33E-04	7.47E-04	3.87E-06	1.26E-03	3.03E-03	2.47E-05	6.37E-04	2.17E-05	2.32E-05	1.62E-03	1.95E-04	6.36E-03	3.21E-04	1.26E-03	1.43E-04	6.56E-04	2.48E-04	1.69E-04	6.74E-04	2.24E-03
		4	2.58E-03	3.66E-05	4.89E-04	7.56E-04	4.30E-06	1.31E-03	2.80E-03	2.38E-05	5.92E-04	2.19E-05	2.23E-05	1.70E-03	2.01E-04	6.27E-03	3.24E-04	1.35E-03	1.40E-04	6.47E-04	2.43E-04	1.67E-04	6.87E-04	2.38E-03
HeLa S3	Rich	1	3.27E-03	9.81E-05	1.12E-03	1.28E-03	4.10E-06	2.80E-03	1.18E-03	3.89E-05	1.34E-03	1.57E-05	9.37E-05	9.36E-04	3.40E-04	8.04E-03	7.28E-04	3.02E-03	9.66E-05	1.43E-03	3.55E-04	3.49E-04	5.64E-04	4.47E-03
		2	3.13E-03	9.70E-05	1.06E-03	1.18E-03	4.91E-06	2.64E-03	1.12E-03	4.24E-05	1.20E-03	2.00E-05	1.14E-04	9.66E-04	3.09E-04	7.35E-03	6.82E-04	2.73E-03	9.35E-05	1.64E-03	3.75E-04	3.83E-04	6.08E-04	4.12E-03
		3	3.27E-03	1.11E-04	1.45E-03	1.28E-03	1.27E-05	2.78E-03	1.21E-03	8.49E-05	1.61E-03	2.49E-05	1.67E-04	9.02E-04	4.04E-04	8.28E-03	6.73E-04	3.15E-03	1.71E-04	2.54E-03	5.54E-04	5.86E-04	8.70E-04	4.55E-03
		4	3.16E-03	8.32E-05	1.17E-03	1.25E-03	3.97E-06	2.76E-03	1.19E-03	4.17E-05	1.10E-03	1.65E-05	8.85E-05	8.00E-04	3.03E-04	8.03E-03	7.02E-04	2.87E-03	1.06E-04	1.47E-03	3.54E-04	3.66E-04	6.13E-04	4.32E-03
		5	3.73E-03	1.14E-04	1.09E-03	1.27E-03	4.37E-06	2.74E-03	1.11E-03	5.39E-05	1.58E-03	2.44E-05	1.27E-04	1.02E-03	3.48E-04	7.57E-03	7.00E-04	2.86E-03	1.13E-04	1.76E-03	4.30E-04	4.61E-04	7.42E-04	4.50E-03
NB4	Rich	1	2.58E-04	1.41E-05	6.04E-04	8.50E-05	4.76E-06	1.66E-04	1.61E-03	1.50E-05	1.70E-04	1.02E-05	5.10E-06	4.91E-04	1.52E-04	3.94E-04	4.25E-05	2.11E-04	4.53E-05	6.55E-05	1.06E-04	5.51E-05	2.23E-04	2.79E-04
		2	4.21E-04	2.95E-05	1.14E-03	1.65E-04	5.36E-06	2.77E-04	2.77E-03	1.44E-05	3.65E-04	1.80E-05	1.14E-05	8.49E-04	2.66E-04	6.58E-04	6.97E-05	3.51E-04	7.32E-05	1.03E-04	1.54E-04	9.00E-05	4.17E-04	4.37E-04
		3	3.30E-04	2.93E-05	9.64E-04	1.27E-04	4.80E-06	2.29E-04	2.37E-03	1.52E-05	2.63E-04	1.42E-05	1.09E-05	7.00E-04	2.36E-04	5.35E-04	5.73E-05	2.80E-04	7.56E-05	1.01E-04	1.38E-04	8.58E-05	3.89E-04	3.69E-04
		4	1.92E-04	1.51E-05	5.53E-04	6.17E-05	2.92E-06	1.40E-04	1.53E-03	1.02E-05	1.15E-04	1.09E-05	5.67E-06	4.42E-04	1.32E-04	3.37E-04	3.79E-05	1.78E-04	5.02E-05	6.61E-05	9.10E-05	5.97E-05	2.28E-04	2.22E-04
MC/CAR	Rich	1	3.22E-03	1.68E-05	4.10E-04	6.64E-04	3.36E-06	1.70E-03	2.85E-03	5.61E-06	2.23E-03	7.42E-06	5.20E-06	2.53E-03	6.92E-04	3.97E-03	3.20E-04	1.61E-03	9.38E-05	1.26E-04	8.17E-05	6.27E-05	3.73E-04	3.34E-03
		2	2.91E-03	1.73E-05	4.47E-04	6.31E-04	3.50E-06	1.63E-03	2.73E-03	5.82E-06	2.31E-03	8.15E-06	6.26E-06	2.15E-03	7.58E-04	3.90E-03	3.07E-04	1.49E-03	1.03E-04	1.39E-04	9.15E-05	7.25E-05	3.99E-04	3.21E-03
		3	3.06E-03	1.46E-05	4.06E-04	6.33E-04	2.84E-06	1.62E-03	2.59E-03	3.71E-06	2.36E-03	7.90E-06	4.84E-06	2.05E-03	7.55E-04	3.66E-03	3.10E-04	1.54E-03	9.08E-05	1.23E-04	7.34E-05	6.44E-05	3.44E-04	3.18E-03
		4	4.36E-03	2.31E-05	4.36E-04	9.06E-04	2.37E-06	2.17E-03	3.58E-03	3.58E-06	2.66E-03	6.82E-06	4.75E-06	2.83E-03	8.24E-04	5.18E-03	4.31E-04	2.10E-03	8.11E-05	1.22E-04	7.36E-05	5.77E-05	3.21E-04	4.28E-03
