# Supplementary material for: Absolute Quantification of the Central Carbon Metabolome in Eight Commonly Applied Prokaryotic and Eukaryotic Model Systems
Source: Metabolites. 2020 Feb 19;10(2):74. doi: 10.3390/metabo10020074 (PMC7073941; doi:10.3390/metabo10020074)
Supplement: Supplementary file 1 [file metabolites-10-00074-s001.zip › TableS3 Rost et al Jan2020.pdf]

Table S3 Contribution of metabolite classes: Total intracellular concentrations (M) and contribution (%) to total listed for each metabolite class and the total of all classes. TCA; tricarboxylic acid, PPP; pentose phosphate pathway.

Organism/ cell line	Culture medium	Total intracellular concentration (M)/ percentage (%)	Metabolite class								Total
			Glycolysis	PPP	Other sugar phosphates	Lactate	TCA cycle	Nucleoside phosphates	Deoxynucleoside phosphates	Amino acids	
Bacillus subtilis	Mineral	M	9.9E-04	3.0E-04	4.1E-04	5.2E-04	3.6E-04	1.9E-03	1.4E-04	2.7E-03	7.3E-03
		%	13.6	4.0	5.6	7.1	4.9	26.6	1.9	36.4	100
	Rich	M	1.3E-03	8.8E-05	2.7E-04	2.6E-04	1.1E-04	1.9E-03	1.5E-04	2.4E-03	6.5E-03
		%	19.5	1.4	4.1	4.1	1.7	29.7	2.3	37.2	100
Saccharomyces cerevisiae	Mineral	M	6.2E-03	2.3E-04	1.1E-03	6.7E-04	2.5E-03	3.9E-03	9.1E-05	1.7E-01	1.8E-01
		%	3.4	0.1	0.6	0.4	1.4	2.2	0.1	91.9	100
	Rich	M	7.0E-03	7.3E-04	1.1E-03	1.1E-03	1.8E-03	4.6E-03	8.9E-05	1.5E-01	1.7E-01
		%	4.1	0.4	0.6	0.6	1.1	2.7	0.1	90.3	100
Nannochloropsis oceanica	Mineral	M	2.6E-03	3.9E-04	7.6E-04	2.3E-03	1.0E-02	2.9E-03	1.7E-05	2.4E-01	2.6E-01
		%	1.0	0.2	0.3	0.9	3.9	1.1	0.0	92.6	100
Phaeodactylum tricornutum	Mineral	M	1.1E-03	6.7E-04	3.8E-04	1.0E-03	1.6E-02	1.2E-03	4.7E-06	5.7E-02	7.7E-02
		%	1.4	0.9	0.5	1.3	20.2	1.6	0.0	74.1	100
Hek293	Rich	M	9.1E-04	5.9E-05	1.4E-03	3.6E-02	2.4E-03	5.5E-03	5.7E-05	1.8E-01	2.2E-01
		%	0.4	0.0	0.6	15.9	1.1	2.5	0.0	79.5	100
HeLa S3	Rich	M	1.8E-03	2.2E-04	2.6E-03	3.3E-02	3.0E-03	5.3E-03	5.4E-05	1.9E-01	2.3E-01
		%	0.8	0.1	1.1	14.3	1.3	2.3	0.0	80.1	100
NB4	Rich	M	4.7E-04	3.3E-05	5.6E-04	1.6E-02	1.7E-03	2.1E-03	3.4E-05	3.0E-02	5.0E-02
		%	0.9	0.1	1.1	31.3	3.5	4.2	0.1	58.8	100
MC/CAR	Rich	M	2.6E-03	1.7E-05	3.4E-04	2.0E-02	2.7E-03	3.3E-03	3.6E-05	8.8E-02	1.2E-01
		%	2.2	0.0	0.3	17.4	2.3	2.8	0.0	74.9	100
