# Supplementary material for: Absolute Quantification of the Central Carbon Metabolome in Eight Commonly Applied Prokaryotic and Eukaryotic Model Systems
Source: Metabolites. 2020 Feb 19;10(2):74. doi: 10.3390/metabo10020074 (PMC7073941; doi:10.3390/metabo10020074)
Supplement: Supplementary file 1 [file Figure S1 Rost at al Feb2020.pdf › Figure S1 Rost at al Feb2020.pdf]

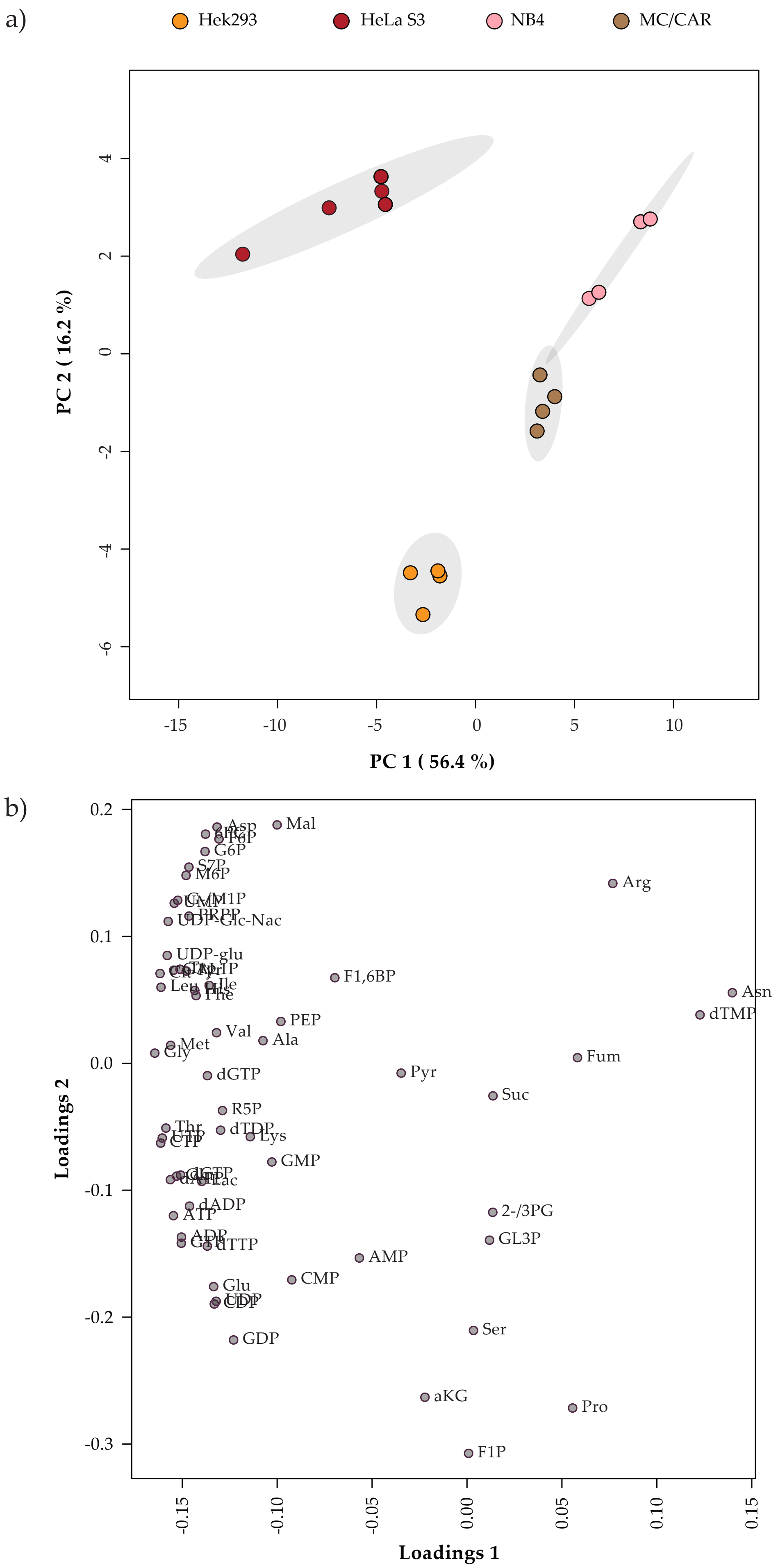

Figure S1 PCA scores and loadings for human cell lines: a) Scores and **b)** loadings plots with 95% confidence intervals from principal component analysis (PCA) of metabolite levels in all biological replicates of the human cell lines Hek393, HeLa S3, NB4, and MC/CAR [48]. Metabolite abbreviations are listed in Table S1.
