# Supplementary material for: Absolute Quantification of the Central Carbon Metabolome in Eight Commonly Applied Prokaryotic and Eukaryotic Model Systems
Source: Metabolites. 2020 Feb 19;10(2):74. doi: 10.3390/metabo10020074 (PMC7073941; doi:10.3390/metabo10020074)
Supplement: Supplementary file 1 [file metabolites-10-00074-s001.zip › TableS5 Rost et al Feb2020.pdf]

Table S5 ***Escherichia coli*** **sampling parameters**: Sampling methods and parameters tested for sampling *E.coli* , listed with resulting energy charge (EC) ± standard deviation (SD) for cultures in rich and mineral medium

Method	Sampling parameters					Average EC ± SD	
						Rich medium	Mineral medium
	**Filter**	**Filter pore size (µm)**	**Vacuum preassure (Below ambient, mbar)**	**Sampling OD_{600}**	**Sampling volume (mL)**		
Filtering	Whatman glass microfiber filter GF/C^{TM} (1822-047, GE Healthcare) stacked on Supor hydrophilic polyethersulfone filter (60110, Pall)	1.2, 0.8	350-500	1	10	0.43 ± 0.10	0.52 ±0.11
	Supor hydrophilic polyethersulfone filter (60110, Pall)	0.8	200	1	2 x 5*	0.61 ± 0.04	0.61 ±0.21
	**Time (min)**	**Temperature (°C)**	**Centrifugal force (x g)**	**Sampling OD_{600}**	**Sampling volume (mL)**		
Cold centrifugation	2 x 5**	4	4500	1	10	0.34 ± 0.14	0.45 ±0.16

* 2 filters, 5 ml/filter to decrease biomass/filter. Filters were consequtivley sampled and pooled into one extract

** Pellets were re-suspended in cold MQ-H_2O between centrifugation steps
