# Supplementary material for: Absolute Quantification of the Central Carbon Metabolome in Eight Commonly Applied Prokaryotic and Eukaryotic Model Systems
Source: Metabolites. 2020 Feb 19;10(2):74. doi: 10.3390/metabo10020074 (PMC7073941; doi:10.3390/metabo10020074)
Supplement: Supplementary file 1 [file metabolites-10-00074-s001.zip › TableS4 Rost et al Feb2020.pdf]

Table S4 YMDB entries: Average (AVG) intracellular concentration (μM) ± standard deviation (SD) of all metabolites included in the panel for metabolite profiling of *Saccharomyces cerevisae* cultured in mineral and rich media, listed with respective Yeast Metabolome Database (YMDB) IDs and range of concentration entries. TCA; tricarboxylic acid, PPP; pentose phosphate pathway. Metabolite abbreviations are listed in Table S1.

Class	Metabolite	YMDB ID	Concentration (μM) Mineral media (AVG±SD)	Concentration (μM) Rich media (AVG±SD)	YMDB (Range)
Glycolysis	G6P	YMDB00947	60,1 ± 14,7	82,4 ± 12,2	2300 ± 200
	F6P	YMDB00078	70,4 ± 17,3	95,3 ± 11,8	650 ± 30
	F1,6BP	YMDB00108	4457,5 ± 1567,8	3710,3 ± 410,6	50-3500 ± 0-1000
	2PG	YMDB00675	715,7 ± 171,2	697,2 ± 57,4	NA
	3PG	YMDB00756	715,7 ± 171,2	697,2 ± 57,4	120-630 ± 10-100
	PEP	YMDB00276	65,2 ± 13,7	85,0 ± 2,9	15 ± 15
	Pyr	YMDB00175	1621,6 ± 163,5	1521,1 ± 204,3	100-5000 ± 0-5000
PPP	6PG	YMDB00218	207,3 ± 57,6	75,6 ± 5,4	200 ± 100
	R5P	YMDB00119	131,0 ± 23,2	34,6 ± 3,3	NA
	S7P	YMDB00752	391,6 ± 83,4	118,0 ± 7,2	NA
Other sugar phosphates	F1P	YMDB00655	48,0 ± 9,4	65,7 ± 13,3	NA
	G1P	YMDB00288	11,9 ± 3,2	13,2 ± 1,5	100 ± 0
	M1P	YMDB00386	11,9 ± 3,2	13,2 ± 1,5	NA
	GAL1P	YMDB00004	17,9 ± 4,2	26,5 ± 3,3	NA
	GL3P	YMDB00073	121,0 ± 15,0	74,5 ± 9,0	NA
	M6P	YMDB00242	37,6 ± 12,1	49,6 ± 4,1	NA
	UDP-glu	YMDB00415	375,1 ± 73,3	397,0 ± 30,5	300 ± 0
	UDP-Glc-Nac	YMDB00277	392,5 ± 72,9	410,8 ± 35,1	NA
	PRPP	YMDB00402	86,9 ± 36,7	68,6 ± 7,6	NA
	Lac	YMDB00247	1050,6 ± 262,1	674,2 ± 97,2	NA
TCA cycle	Cit	YMDB00086	766,7 ± 182,0	775,1 ± 61,6	200-13500 ± 0-9500
	Icit	YMDB00026	10,6 ± 3,1	38,7 ± 6,7	NA
	aKG	YMDB00153	117,6 ± 22,2	649,9 ± 103,6	10-5500 ± 0-2300
	Suc	YMDB00338	493,5 ± 85,9	555,9 ± 70,9	600 ± 130
	Fum	YMDB00101	69,2 ± 7,7	74,8 ± 2,1	NA
	Mal	YMDB00280	330,9 ± 51,9	388,5 ± 13,8	2515 ± 126
Nucleoside phosphates	AMP	YMDB00097	222,6 ± 40,2	254,1 ± 26,1	100-600 ± 20-300
	ADP	YMDB00914	839,6 ± 160,1	657,3 ± 42,3	300-1400 ± 20-800
	ATP	YMDB00109	1795,3 ± 322,1	1183,4 ± 105,8	1200-2800 ± 100-1750
	GMP	YMDB00261	48,6 ± 10,8	75,4 ± 8,5	NA
	GDP	YMDB00257	166,1 ± 33,2	186,1 ± 25,0	NA
	GTP	YMDB00558	465,4 ± 90,3	420,0 ± 29,5	NA
	CMP	YMDB00406	13,0 ± 4,2	22,2 ± 4,5	NA
	CDP	YMDB00064	74,4 ± 22,7	79,1 ± 8,3	NA
	CTP	YMDB00279	324,8 ± 59,3	259,1 ± 26,2	NA
	UMP	YMDB00049	175,4 ± 31,5	192,2 ± 12,9	NA
	UDP	YMDB00307	87,8 ± 25,7	129,2 ± 11,7	NA
	UTP	YMDB00326	415,4 ± 78,5	438,2 ± 41,2	NA
Deoxynucleotides	dAMP	YMDB00451	0,5 ± 0,1	1,0 ± 0,2	NA
	dADP	YMDB00901	22,5 ± 4,8	25,7 ± 2,2	NA
	dATP	YMDB00700	23,3 ± 3,8	21,9 ± 1,5	44 ± 0
	dGTP	YMDB00744	8,4 ± 1,8	7,9 ± 0,5	18 ± 0
	dCTP	YMDB00658	9,2 ± 1,9	8,3 ± 0,7	16 ± 0
	dUMP	YMDB00221	0,2 ± 0,0	<LOQ	NA
	dTMP	YMDB00162	1,4 ± 0,2	1,5 ± 0,1	NA
	dTDP	YMDB00205	6,6 ± 2,1	9,3 ± 1,1	NA
	dTTP	YMDB00745	17,2 ± 3,0	15,8 ± 1,6	70 ± 0
Amino acids	Ala	YMDB00154	9339,6 ± 1493,8	9541,6 ± 1269,4	6000-19000 ± 100-3000
	Arg	YMDB00592	12410,1 ± 2146,4	10230,7 ± 819,2	2000-190000 ± 0-7000
	Asn	YMDB00226	1252,2 ± 206,4	1745,5 ± 316,3	400-800 ± 20-120
	Asp	YMDB00896	12560,1 ± 1857,0	24020,5 ± 1216,5	600-8000 ± 10-5000
	Gln	YMDB00002	37294,5 ± 4816,5	5461,9 ± 390,5	15000-17000 ± 0-2000
	Glu	YMDB00271	61334,9 ± 8852,3	64614,4 ± 3260,6	1200-80000 ± 20-12500
	Gly	YMDB00016	2039,8 ± 233,1	3213,6 ± 380,2	780-15000 ± 0-145
	His	YMDB00369	1744,3 ± 226,7	3918,2 ± 218,4	800-9000 ± 40-200
	Ile	YMDB00038	537,6 ± 62,9	2071,0 ± 677,4	150-6200 ± 3-120
	Leu	YMDB00387	595,2 ± 70,5	10950,3 ± 3489,0	235-2700 ± 5-150
	Lys	YMDB00330	3591,6 ± 672,4	11489,7 ± 1602,5	300-3600 ± 5-200
	Met	YMDB00318	141,0 ± 16,3	1280,2 ± 325,2	50-600 ± 1-30
	Phe	YMDB00304	378,2 ± 36,8	2428,5 ± 766,4	186-3000 ± 4-70
	Pro	YMDB00378	729,0 ± 91,3	1729,4 ± 180,4	1075 ± 54
	Ser	YMDB00112	3276,2 ± 445,5	4368,8 ± 532,1	293-5800 ± 6-115
	Thr	YMDB00214	3253,2 ± 392,3	4570,4 ± 473,1	600-4200 ± 10-200
	Trp	YMDB00126	65,4 ± 6,6	467,3 ± 222,2	240-450 ± 20-70
	Tyr	YMDB00364	299,3 ± 36,5	528,6 ± 173,5	170-4000 ± 6-80
	Val	YMDB00152	2061,7 ± 282,5	3040,1 ± 814,8	525-2800 ± 10-140
